# The NTE domain of PTENα/β promotes cancer progression by interacting with WDR5 via its SSSRRSS motif

**DOI:** 10.1038/s41419-024-06714-6

**Published:** 2024-05-14

**Authors:** Xiaolei Huang, Cheng Zhang, Xinci Shang, Yichang Chen, Qin Xiao, Zhengguo Wei, Guanghui Wang, Xuechu Zhen, Guoqiang Xu, Jinrong Min, Shaoming Shen, Yanli Liu

**Affiliations:** 1 Jiangsu Key Laboratory of Neuropsychiatric Diseases, Jiangsu Province Engineering Research Center of Precision Diagnostics and Therapeutics Development, College of Pharmaceutical Sciences, Soochow University, 215123 Suzhou, Jiangsu China; 2 Institute of Aging & Tissue Regeneration, Ren-Ji Hospital, Shanghai Jiao Tong University School of Medicine (SJTU-SM), 200127 Shanghai, China; 3 School of Biology and Basic Medical Science, Soochow University, 215123 Suzhou, Jiangsu China; 4 Hubei Key Laboratory of Genetic Regulation and Integrative Biology, School of Life Sciences, Central China Normal University, 430079 Wuhan, Hubei China

**Keywords:** Nanocrystallography, Liver cancer

## Abstract

PTENα/β, two variants of PTEN, play a key role in promoting tumor growth by interacting with WDR5 through their N-terminal extensions (NTEs). This interaction facilitates the recruitment of the SET1/MLL methyltransferase complex, resulting in histone H3K4 trimethylation and upregulation of oncogenes such as *NOTCH3*, which in turn promotes tumor growth. However, the molecular mechanism underlying this interaction has remained elusive. In this study, we determined the first crystal structure of PTENα-NTE in complex with WDR5, which reveals that PTENα utilizes a unique binding motif of a sequence SSSRRSS found in the NTE domain of PTENα/β to specifically bind to the WIN site of WDR5. Disruption of this interaction significantly impedes cell proliferation and tumor growth, highlighting the potential of the WIN site inhibitors of WDR5 as a way of therapeutic intervention of the PTENα/β associated cancers. These findings not only shed light on the important role of the PTENα/β-WDR5 interaction in carcinogenesis, but also present a promising avenue for developing cancer treatments that target this pathway.

## Introduction

PTEN (phosphatase and tensin homolog) is a tumor suppressor protein comprised of 403 amino acids, which was initially identified in 1997 by various research groups exploring the cancer susceptibility locus on human chromosome 10q23 [1–3]. Mutations of PTEN have been linked to various cancers, including liver, prostate, and breast cancer [1–3]. Over 80% of Cowden syndrome patients carry PTEN mutations, which are primarily characterized by hamartomas [4]. PTEN exhibits both protein phosphatase and lipid phosphatase activities, so it can catalyze the dephosphorylation of p-Tyr, p-Ser, and p-Thr residues in proteins, as well as the phosphoinositide lipid at position D3 of PtdIns(3,4,5)P3, PIP3 [5, 6]. By dephosphorylating PIP3, PTEN negatively regulates the PI3K/AKT pathway, which is crucial for cell survival and proliferation. Loss of the PTEN function results in a constitutionally activated PI3K/AKT pathway, promoting cell growth, proliferation, and survival while inhibiting apoptosis [7, 8].

Recent studies have identified multiple non-AUG initiation codons located within its 5’ untranslated region of the PTEN mRNA [9–12]. This discovery has led to the identification of various isoforms of the canonical PTEN protein with different lengths of extra amino acids at their N-terminus [9–12]. Specifically, three variants known as PTENα (also called PTEN-L) [9, 12], PTENβ [10], and PTENε [11] have been discovered, which are translated at 519 bp, 438 bp, and 216 bp upstream of the AUG start codon, respectively, and add 173, 146, and 72 amino acids to the N-terminus of the canonical PTEN protein as an N-terminal extension (NTE) domain. Bioinformatic analysis has also revealed that the NTE region contains an intrinsically disordered region (IDR) that is rich in polar residues, potential linear binding motifs, protein-binding sites, and post-translational modification (PTM) sites. These findings suggest that the NTE may serve as a signaling platform in regulating the biological functions and intracellular trafficking of PTEN [13].

The initial study on PTENα revealed that it possesses a secretion signal sequence and a resembled cell-penetrating element, which enable it to be secreted out of cells and taken up by other cells. This ability allows PTENα to inhibit the growth of glioblastoma tumors by interfering with the PI3K signaling pathway [9]. Meanwhile, another study demonstrated that PTENα is localized in mitochondria, where it regulates energy metabolism [12]. In contrast, PTENβ which was initially found in the nucleus negatively regulates nucleolin phosphorylation levels, pre-RNA synthesis and cell proliferation [10]. On the other hand, recent research has shown that both PTENα and PTENβ (without the secretion signal sequence) can be secreted into the extracellular space and cleaved by the proprotein convertase Furin, yielding a C-terminal fragment that significantly suppresses the proliferation of tumor cells [14]. Besides being present in the extracellular space, PTENα and PTENβ are also prominently localized in the nucleus and promote liver cancer cell growth by interacting with WDR5 protein through their NTEs [15]. PTENε, a smaller PTEN variant primarily located in the cell plasma membrane, inhibits the formation of pseudopods and reduces the migratory ability of tumor cells, thereby repressing cancer cell metastasis [11]. Overall, these findings emphasize the crucial roles played by PTENα/β/ε in maintaining normal cell survival and inhibiting or promoting tumor cell proliferation and migration, underscoring the complexity of their involvement in tumor progression.

WDR5 is a highly conserved protein with a seven-bladed β-propeller fold. It is an essential subunit of the SET1/MLL methyltransferase complexes, which regulate gene expression by catalyzing methylation at histone H3K4 sites [16, 17]. WDR5 performs its role by presenting H3K4 for methylation, through the recognition of the guanidyl group of H3R2 by utilizing its central channel of the β-propeller structure located at the top face [18]. This region is known as WDR5 interacting site or the WIN site [19]. Apart from H3R2, WDR5 also interacts with other arginine-containing sequences called the WIN motif from the catalytic component of the SET1/MLL complexes [19–21] and other proteins [22]. In addition to the WIN site, WDR5 also interacts with the MbIIIb motif of MYC, a well-known oncoprotein, through its WBM (WDR5 binding motif) site [23]. Due to its unique epigenetic role by means of interacting with various ligands, WDR5 is involved in many biological processes, including reproduction [24, 25], development [26–28], metabolism [29], immune responses and inflammation [30–32], neural and humoral regulation [33, 34]. Additionally, WDR5 is implicated in the onset, progression, and maintenance of multiple diseases. For instance, overexpression of WDR5 is not only associated with the development and progression of various cancers, such as prostate cancer [35, 36], breast cancer [37], leukemia [38], and liver cancer [39], but also linked to unfavorable clinical outcomes. Both the WIN site and WBM site are involved in cancer progression [40, 41]. Consequently, it appears to be a promising approach for treatment of these diseases by targeting WDR5.

The previous study has shown that PTENα/β promotes tumor progression in liver cancer cells by interacting with WDR5 to recruit the SET1/MLL complexes to the promoters of the PTENα/β-target genes, such as *NOTCH3*, *SLC12A5* and *TCF19*, the broadly studied oncogenes [15, 42–45]. Therefore, understanding the molecular mechanism of the interactions between PTENα/β and WDR5, and deciphering its underlying molecular functions hold substantial importance for exploring potential therapeutic applications. In this study, we aimed to investigate the structural details of the interactions between PTENα/β-NTE and WDR5 using biophysical binding assays and X-ray crystal structure analysis. We discovered that PTENα/β-NTE utilizes an N-terminal WIN motif to interact with the arginine-binding pocket of WDR5. Interestingly, we identified a unique -RR- motif in PTENα/β-NTE for binding to the WDR5 WIN site, which differs from the conserved -A/CR- motif found in other WIN motif sequences. To validate the functional significance of this interaction, cell and xenograft mouse experiments were conducted, which demonstrated that disrupting the PTENα-WDR5 interaction by point mutations reduces the expression of downstream oncogenes, inhibiting cell proliferation and tumor growth. Our findings uncover the molecular mechanism of how PTENα/β-NTE functions, provide the valuable insights into the crucial role of PTENα/β-NTE in cancer biology, and offer potential therapeutic targets for cancer treatment.

## Results

### The N-terminal extension (NTE) of PTENα/β binds to WDR5

**Fig. 1:**
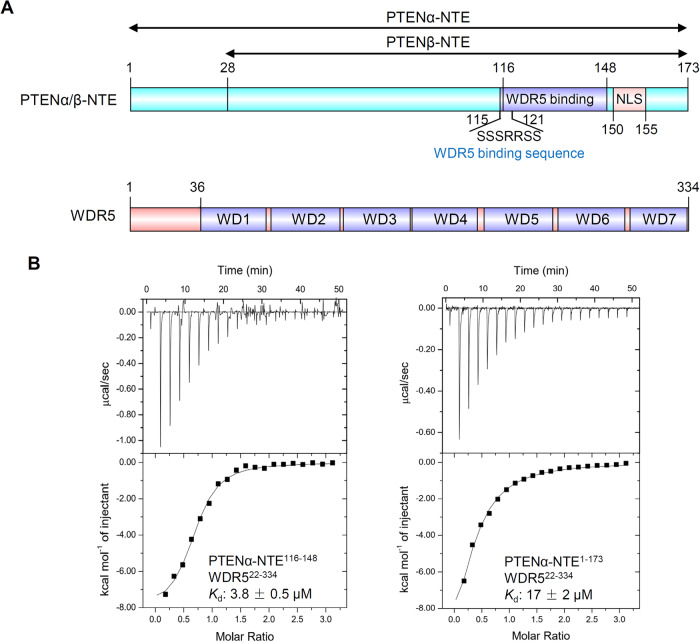
PTENα/β-NTE binds to WDR5. **A** Domain structure of human PTENα/β-NTE and WDR5. PTENα/β-NTE consists of a WDR5 binding motif, a nuclear localization signal (NLS). WDR5 consists of seven WD40 repeats. The WDR5 binding sequence, namely SSSRRSS, under the PTENα/β-NTE was identified by this study. **B** ITC binding curves for the titration of PTENα-NTE^116–148^ or PTENα-NTE^1–173^ to WDR5^22–334^ by iTC-200 microcalorimeter (MicroCal, Inc.). *K*_d_: dissociation constants (μM); ITC data shown are representative of two independent experiments and all *K*_d_ values were calculated from a single measurement and errors were estimated by fitting curve.

A previous study has revealed that PTENα/β-NTE requires two crucial motifs for their tumorigenic activity, a WDR5 binding motif (amino acids 116–148) and an essential nuclear localization signal (NLS) (Fig. 1A) [15]. To quantify the interaction between PTENα/β-NTE and WDR5, isothermal titration calorimetry (ITC) assays were conducted to determine the dissociation constants (*K*_d_) of WDR5^22–334^ with a synthetic PTENα-NTE^116–148^ peptide and the recombinant PTENα-NTE^1–173^ protein. The ITC data indicated that both the PTENα-NTE^116–148^ peptide and the recombinant PTENα-NTE^1–173^ protein could bind to WDR5^22–334^, with the former exhibiting a higher binding affinity than the latter (*K*_d_: 3.8 μM *vs*. 17 μM, Fig. 1B). This suggested that the PTENα-NTE^116–148^ fragment is sufficient for their interaction in vitro. The reason for the higher binding capacity of PTENα-NTE^116–148^ may be due to a shorter peptide fragment without irrelevant parts to disturb the interaction, which is consistent with the previous study of WDR5 and LANA (*K*_d_: 25 μM *vs*. 2.3 μM for LANA^16–32^ and LANA^19–32^ bound to WDR5 as determined by ITC, respectively) [46]. Our ITC data provide evidence supporting the fact that PTENα/β-NTE directly binds to WDR5 in vitro, since the PTENα-NTE^116–148^ fragment is a common region for both NTEs of the two PTEN variant proteins.

### PTENα/β-NTE binds to the WIN site of WDR5 by its SSSRRSS motif

**Fig. 2:**
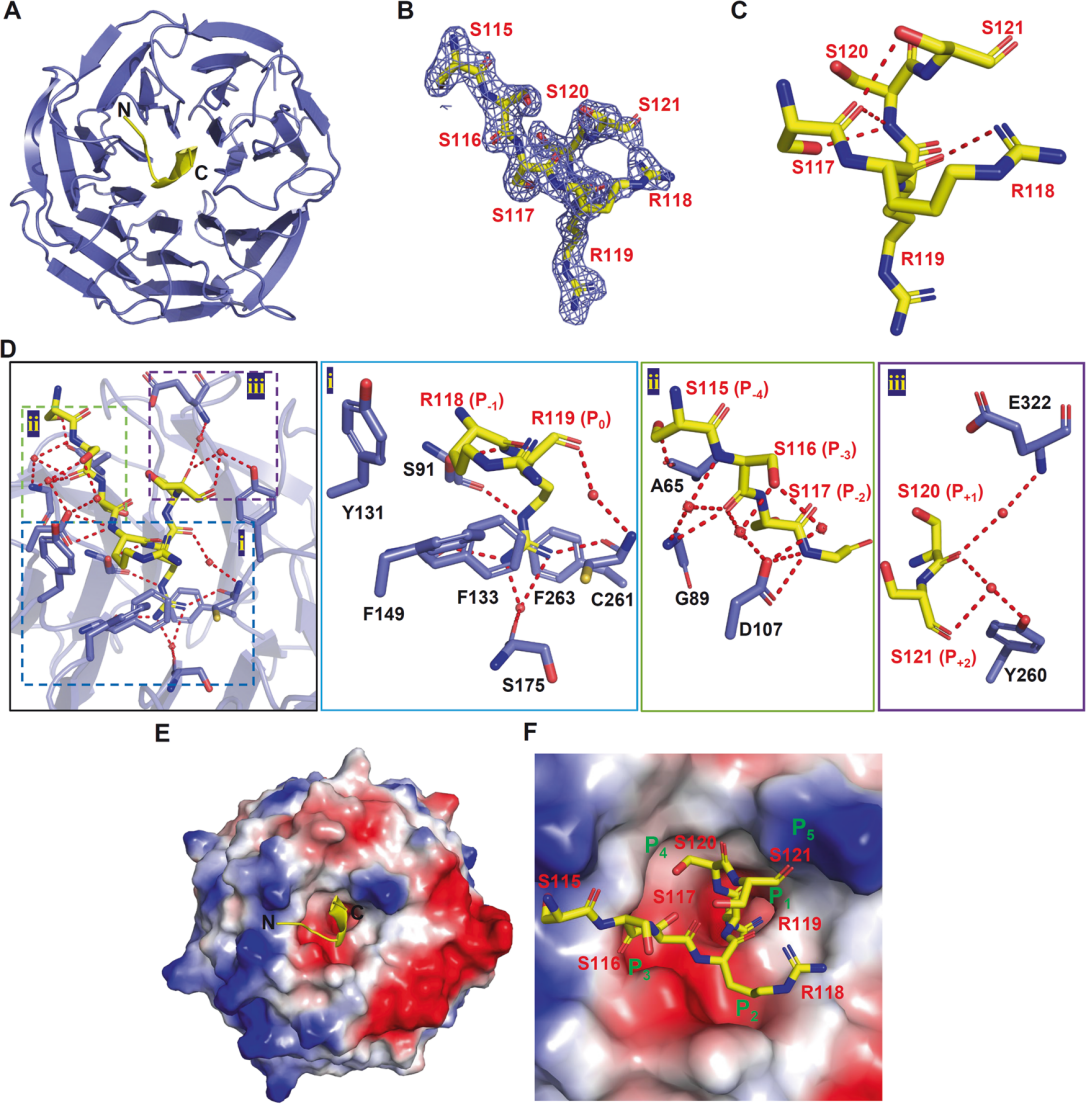
Crystal structure of WDR5^22–334^ in complex with PTENα-NTE^1–173^. **A** Overall structure of WDR5 in complex with PTENα-NTE. The structure was shown in cartoon with WDR5 colored in slate and PTENα-NTE colored in yellow. **B** Fo-Fc omit map of PTENα-NTE contoured at 1σ level. **C** Intramolecular hydrogen bonds stabilize the WDR5-PTENα-NTE interaction. **D** Detailed interaction between PTENα-NTE and WDR5. Amino acid residues of WDR5 involved in the PTENα-NTE interaction were shown as sticks and the detailed interactions were shown by enlarged views. Key hydrogen bonds were depicted as red dash lines and key water molecules were indicated as red sphere. **E**, **F** Electrostatic potential surface view of WDR5 in complex with PTENα-NTE. The PTENα-NTE was shown as cartoon (**E**) and sticks (**F**), respectively. Five WIN motif binding pockets (P_1_–P_5_) in WDR5 were labeled with green (**F**).

To better understand how PTENα/β and WDR5 interact at the molecular level, we attempted to crystallize WDR5 in complex with the recombinant PTENα-NTE^1–173^ protein or the synthetic PTENα-NTE^116–148^ peptide and successfully solved the crystal structures of WDR5 bound and unbound by PTENα-NTE^1–173^ (Fig. 2, Supplementary Fig. S1 and Table 1). Initially, we attempted to co-crystallize the synthetic PTENα-NTE^116–148^ peptide with full-length WDR5 (amino acids 1–334), but only obtained the crystal structure of WDR5 in the PTENα-NTE peptide-free form at 1.8 Å resolution. In this apo WDR5 structure, the N-terminal residues (^11^EAARAQPT^18^) of WDR5 occupy the WIN site, with WDR5-R14 inserting into the negatively charged channel of the WIN site, as previous studies have shown [18, 46] (Supplementary Fig. S1). Therefore, we tried using a WDR5^22–334^ truncation for co-crystallization screening with PTENα-NTE^116–148^ or PTENα-NTE^1–173^ and eventually obtained the co-crystals of WDR5^22–334^ with PTENα-NTE^1–173^.

**Table 1:** Data collection and refinement statistics.

	WDR5-PTEN	WDR5
**PDB code**	8X3S	8X3R
**Data collection**		
Space group	P 2_1_ 2_1_ 2_1_	P 2_1_ 2_1_ 2_1_
Cell dimensions		
* a, b, c* (Å)	46.5, 63.8, 92.8	48.6, 52.7, 123.1
* α, β, γ* (◦)	90, 90, 90	90, 90, 90
Resolution (Å)	29.19–1.87 (1.91–1.87)	24.21–1.76 (1.79–1.76)
Measured reflections	40296 (1831)	55450 (2918)
Unique reflections	22704 (1171)	30780 (1719)
*R*_merge_	0.071 (0.533)	0.045 (0.428)
*I/σI*	10.5 (1.8)	10.9 (1.7)
CC_1/2_	0.994 (0.627)	0.998 (0.674)
Completeness (%)	97.0 (80.3)	96.9 (96.6)
Redundancy	1.8 (1.6)	1.8 (1.7)
**Refinement**		
Resolution (Å)	29.19–1.87 (1.94–1.87)	24.21–1.76 (1.82–1.76)
*R*_work_/*R*_free_ (%)	22.4/25.7	19.7/21.2
No. of atoms/average *B*-factors (Å^2^)	2558/33.2	2604/19.9
Protein	2377/32.6	2350/19.6
Ligand	52/36.1	65/23.2
Water	129/37.0	189/20.0
Root mean square deviation		
Bond lengths (Å)	0.01	0.01
Bond angles (°)	1.3	1.2

Values in parentheses are for the highest resolution shell.

The complex structure revealed that WDR5 exhibits the typical seven-bladed β-propeller structure, while PTENα-NTE adopts a 3_10_-helical conformation fitting into the central WIN site pocket of WDR5 (Fig. 2A). Based on the electron density, the residues 115 to 121 of PTENα-NTE could be distinctly traced and form numerous intramolecular hydrogen bonds to compact its conformation (Fig. 2B, C). The interaction between PTENα-NTE and WDR5 involves a network of hydrogen bonds, van der Waals contacts, hydrophobic packing, and cation-π interactions, which are observed in other WDR5 complexes involving the accommodation of the WIN motif ligands within the WIN site (Fig. 2D–F) [18, 20, 21]. Specifically, the key R119 (P_0_) residue of PTENα-NTE extends into the pocket 1 of WDR5, interacting with F133 and F263 side chains through cation-π interaction, as well as making several direct or water-mediated hydrogen bonds with the main chains of S91, F133, S175, and C261 to further stabilize the interaction (Fig. 2D-i, F). While the PTENα-R118 (P_-1_) residue is embraced by pocket 2 via hydrophobic packing with Y131 and F149 residues of WDR5 (Fig. 2D-i, F). The S115–S117 (P_-4 to -2_) residues of PTENα-NTE bind to pocket 3 and form several main-chain and side-chain hydrogen bonds with A65, G89, and D107 residues of WDR5 (Fig. 2D-ii, F). The C-terminus of the PTENα-NTE^115–121^ fragment, comprising S120 (P_+1_) and S121 (P_+2_) residues, is held by pockets 4 and 5 through direct or water-mediated hydrogen bonds with E322 and Y260 residues of WDR5 (Fig. 2D-iii, F). The PTENβ-NTE is expected to bind to WDR5 in the same way as PTENα-NTE since PTENα-NTE^115–121^ fragment is a common region for both of them. Overall, our complex structural studies demonstrated that PTENα/β-NTE binds to the WIN site of WDR5 snugly by forming numerous intermolecular interactions.

### PTENα/β-NTE interacts with WDR5 exclusively through the WIN site

**Fig. 3:**
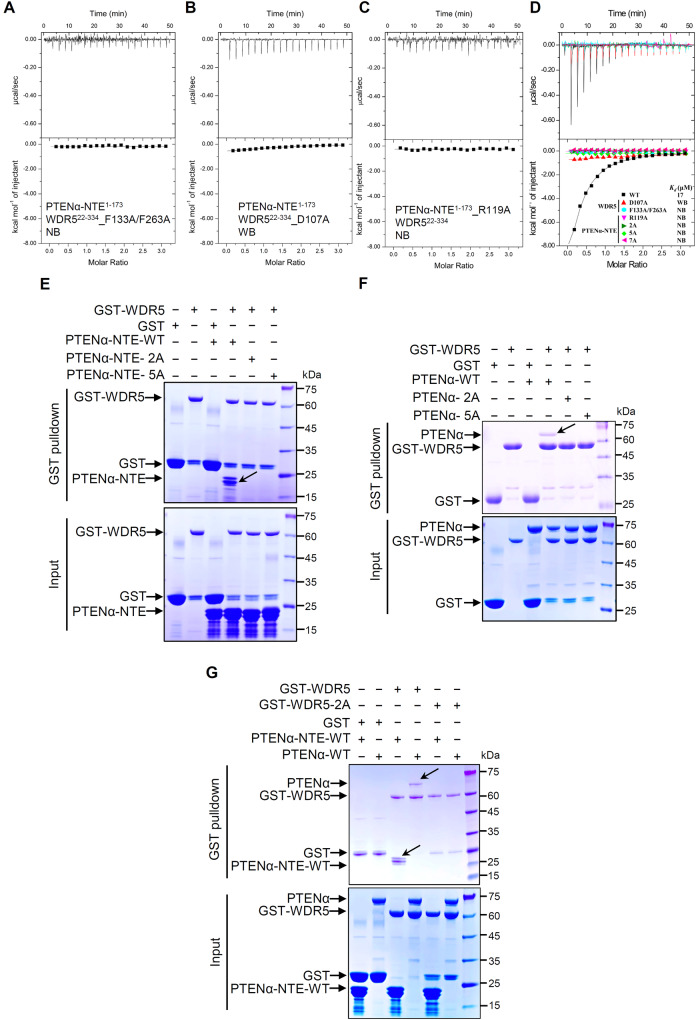
PTENα/β-NTE interacts with the WDR5 WIN site solely through the SSSRRSS WIN motif. **A**–**D** ITC binding curves for the titration of wild-type PTENα-NTE to different mutants of WDR5 (**A**, **B**) or different mutants of PTENα-NTE to wild-type WDR5 (**C**, **D**) by iTC-200 microcalorimeter (MicroCal, Inc.). **E**–**G** Mutation of the interacting residues affected the interaction between PTENα-NTE^1–173^ or full-length PTENα^1–576^ and WDR5^22–334^. In vitro GST pulldown of wild-type WDR5 with wild-type or mutant of PTENα-NTE (**E**) or full-length PTENα (**F**), and wild-type or F133A/263A (WDR5-2A) mutant WDR5 with wild-type PTENα-NTE and full-length PTENα (**G**). Bacterially expressed proteins PTENα-NTE (**E**, **G**) or full length PTENα (**F**, **G**) and their mutants, as indicated, were incubated with GST or GST-tagged WDR5, followed by GST pulldown and CBB staining, with the specific binding indicated by an arrow. *K*_d_: dissociation constants (μM); NB: no detectable binding; WB: weak binding; 2A: PTENα-NTE_R118A/R119A; 5A: PTENα-NTE_115–119-5A; 7A: PTENα-NTE_115–119-5A_R135A/R144A; CBB Coomassie brilliant blue.

To verify our findings from the complex crystal structure, we introduced specific point mutations into WDR5^22–334^ for binding studies. The introduction of the double point mutations WDR5_F133A/263A (referred to as WDR5-2A) into WDR5^22–334^ resulted in the complete loss of the interaction between WDR5 and PTENα-NTE (Fig. 3A), which is consistent with the crucial role played by these two residues in WIN site binding, as indicated by previous studies [47–49]. Additionally, when we introduced the D107A mutation into WDR5^22–334^, another critical residue for WIN site binding, the capacity of WDR5^22–334^ to bind to PTENα-NTE^1–173^ was significantly reduced (Fig. 3B), which also aligns with previous studies [47–49]. Collectively, all these mutants binding assays confirmed that WDR5 recognizes PTENα/β-NTE specifically through the WIN binding site.

To further confirm whether PTENα-NTE^1–173^ interacts with WDR5 only through the WIN binding site, a competitive binding assay was conducted using unmodified histone H3 peptide with residues 1–15 (H3 ^1–15^), a well-known ligand of the WDR5 WIN site [18, 48]. ITC experiments were performed with PTENα-NTE^116–148^ and PTENα-NTE^1–173^ against a mixture of WDR5^22–334^ and H3 ^1–15^ with a 1:1 molar ratio. The results revealed that PTENα-NTE^116–148^ weakly bound to the WDR5^22–334^ and H3 ^1–15^ mixture, with a *K*_d_ of 32 ± 3 μM (Supplementary Fig. S2A). However, no significant binding tendency was observed for PTENα-NTE^1–173^, which may be due to PTENα-NTE^116–148^ having a higher binding affinity for WDR5^22–334^ than H3 ^1–15^, while PTENα-NTE^1–173^ and H3 ^1–15^ having a comparable binding affinity for WDR5^22–334^ (*K*_d_ values: 3.8 ± 0.5, 17 ± 2, and 25 ± 3 μM for PTENα-NTE^116–148^, PTENα-NTE^1–173^, and H3 ^1–15^, respectively, Supplementary Fig. S2A–C). Reverse competitive titration experiments using H3 ^1–15^ against a mixture of WDR5^22–334^ with PTENα-NTE^1–173^ or PTENα-NTE^116–148^ were also conducted, and no notable binding was observed (Supplementary Fig. S2C). Overall, all these binding assays support the conclusion that PTENα/β-NTE interacts with WDR5 exclusively through the WIN site.

### PTENα/β-NTE interacts with the WDR5 WIN site solely through the SSSRRSS WIN motif

To investigate the significance of the ^115^SSSRRSS^121^ WIN motif within PTENα-NTE^1–173^ in binding to wild-type WDR5^22–334^, a series of ITC assays were conducted using various PTENα-NTE^1–173^ mutants. Initially, experiments were carried out using 6×His-TEV (Tobacco etch virus)-PTENα-NTE^1–173^ fusion proteins for ease of purification. Previous reports and our complex structure have highlighted the arginine residue at P_0_ position as a major structural determinant for the WDR5 binding [18, 21]. Therefore, to avoid any possible compensatory effect between R118 (P_-1_) and R119 (P_0_) (Supplementary Fig. S3A), a double point mutant (PTENα-NTE^1–173^-2A: PTENα-NTE^1–173^_R118A/R119A) was tested, which displayed weak binding to WDR5^22–334^ (Supplementary Fig. S3B). Furthermore, the PTENα-NTE^1–173^_115–119-5A mutant (PTENα-NTE^1–173^-5A), and the PTENα-NTE^1–173^-5A_R135A/R144A (PTENα-NTE^1–173^-7A) mutant with mutation of all the arginine residues within PTENα-NTE^116–148^ were generated, which still displayed weaker interactions with WDR5 (Supplementary Fig. S3A, B).

Subsequent literature research identified that the arginine residue within the 6×His-TEV-tag can mimic the WIN motif and bind to the WDR5 WIN site [18], explaining why the tagged PTENα-NTE^1–173^-7A mutant still retained some binding to WDR5. Therefore, all mutant ITC assays were repeated with the His-TEV-tag removed proteins, and the results revealed that none of the mutants bound to WDR5^22–334^ (Supplementary Fig. S3C). Moreover, even a single PTENα-NTE^1–173^-R119A mutant was sufficient to abolish its binding to WDR5^22–334^ (Fig. 3C, D, Supplementary Fig. S3C), indicating that the R119 of PTENα is critical for the interaction between PTENα/β-NTE and WDR5. Finally, the GST pulldown assay demonstrated that WDR5^22–334^ directly and specifically bound to both wild-type PTENα-NTE^1–173^ and full-length PTENα^1–576^ proteins in vitro, but not the mutants containing the R119A (Fig. 3E, F), and the PTENα-NTE^1–173^ and PTENα^1–576^ proteins bound to wild-type WDR5^22–334^ only, but not the mutant containing F133A/263 A (WDR5-2A), neither (Fig. 3G). These findings strongly support the conclusion that PTENα/β-NTE interacts with WDR5 WIN site solely through the SSSRRSS WIN motif.

### A novel -RR- binding motif was identified in PTENα/β-NTE for binding to WDR5

**Fig. 4:**
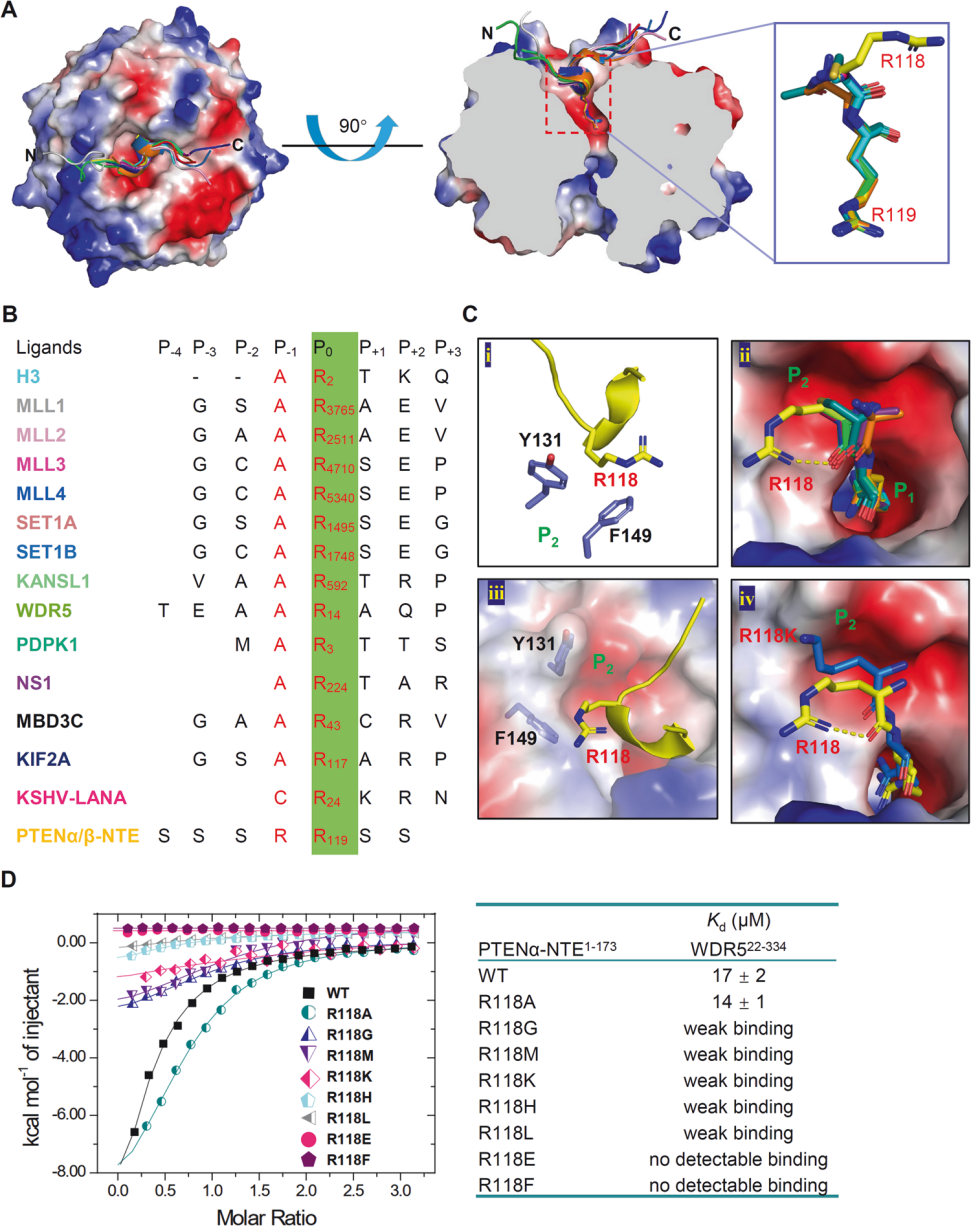
Structural comparison with other WIN site ligands. **A** Different WIN motif ligand peptides bind to WDR5 using the same arginine-binding pocket. Superposition of WIN motif peptides shown with a schematic representation **(left)**, cut-away view of the arginine-binding pocket of WDR5 **(middle)**, and zoomed view of the conserved WIN motif residues (P_0_ and P_-1_) **(right)**. The WDR5 molecule was presented as electrostatic potential surface and the different WIN motif peptides were presented as cartoon diagram by different colors. **B** PTENα/β-NTE has a unique -RR- WIN site binding motif. Sequence alignment of the WIN motif peptides displayed in the complex structures. The key conserved arginine residue was highlighted in green and the conserved WIN motif residues (P_0_ and P_-1_) were represented in red character. **C** The unique R118 residue of the PTENα-NTE binds to the P_2_ pocket of WDR5. **D** Mutations of the PTENα-NTE-R118 residue affected binding to WDR5. ITC curves **(left)** and binding affinities **(right)** for the titration of wild-type or different R118 mutants of PTENα-NTE^1–173^ to WDR5^22–334^.

Comparison with the published WDR5-WIN motif complex structures revealed that all the WIN motifs bind to WDR5 similarly through its conserved arginine residue inserting into the central tunnel of WDR5 and the residue at P_-1_ position displays a preference for a small side-chain residue (Fig. 4A and Supplementary Fig. S4). However, the WIN motif of PTENα/β-NTE, namely the SSSRRSS fragment, bears a large side-chain residue, arginine, at the P_-1_ position, different from the previous reported WIN site ligands with smaller side-chain residues, such as alanine [20, 21, 50–52] and cysteine [46] (Fig. 4A, B and Supplementary Fig. S5). The side chain of R118 of PTENα-NTE^1–173^ extends into another pocket (P_2_ pocket) and forms hydrophobic packing with Y131 and F149 side chains of WDR5 (Fig. 4C-i). To investigate the impact of amino acid identity at position P_-1_ on binding affinity, ITC experiments were performed using various PTENα-NTE^1–173^-R118 mutants (Fig. 4D and Supplementary Fig. S6). As shown in Fig. 4D, all the mutants except R118A weakened the binding due to steric hindrance or lack of enough hydrophobic interaction. Among these mutants, mutating arginine to lysine, which has a similar size and charge to arginine, resulted in weak binding to WDR5^22–334^ too. Further careful structural analysis revealed that an intramolecular hydrogen bond between the side-chain guanidyl group and the main-chain carbonyl group of R118 allows the side chain of R118 to orient closer to the main chain, eliminating spatial hindrance of its insertion into the P_2_ pocket (Fig. 4C-ii). Consequently, the bent side chain of R118 finds a perfect fit within the P_2_ pocket (Fig. 4C-iii), while the side chain of lysine could not form this intramolecular hydrogen bond and may cause spatial hindrance (Fig. 4C-iv). To further validate if arginine at P_-1_ position is generally acceptable for other WDR5 WIN site binding partners, the binding abilities of wild-type MLL1 WIN motif MLL1^3762–3772^ (another well-known ligand of the WDR5 WIN site [19–21]) and its mutant MLL1^3762–3772^_A3764R to WDR5^22–334^ were determined by ITC. As shown in Supplementary Fig. S7, both the wild-type and the mutant MLL1^3762–3772^ bound to WDR5^22–334^ similarly, which indicated that the arginine at P_-1_ position of WIN motif should be generally acceptable to all WIN-motif-containing proteins. Overall, these structural and binding studies highlight the importance of the amino acid identity at the P_-1_ position to the binding, and the novel finding that an arginine residue at the P_-1_ position exhibits robust binding affinity will broaden the ligand repertoire, providing the chance to search for additional WIN site binding partners.

### Mutation of the SSSRRSS motif of PTENα-NTE diminishes expression of PTENα target genes

**Fig. 5:**
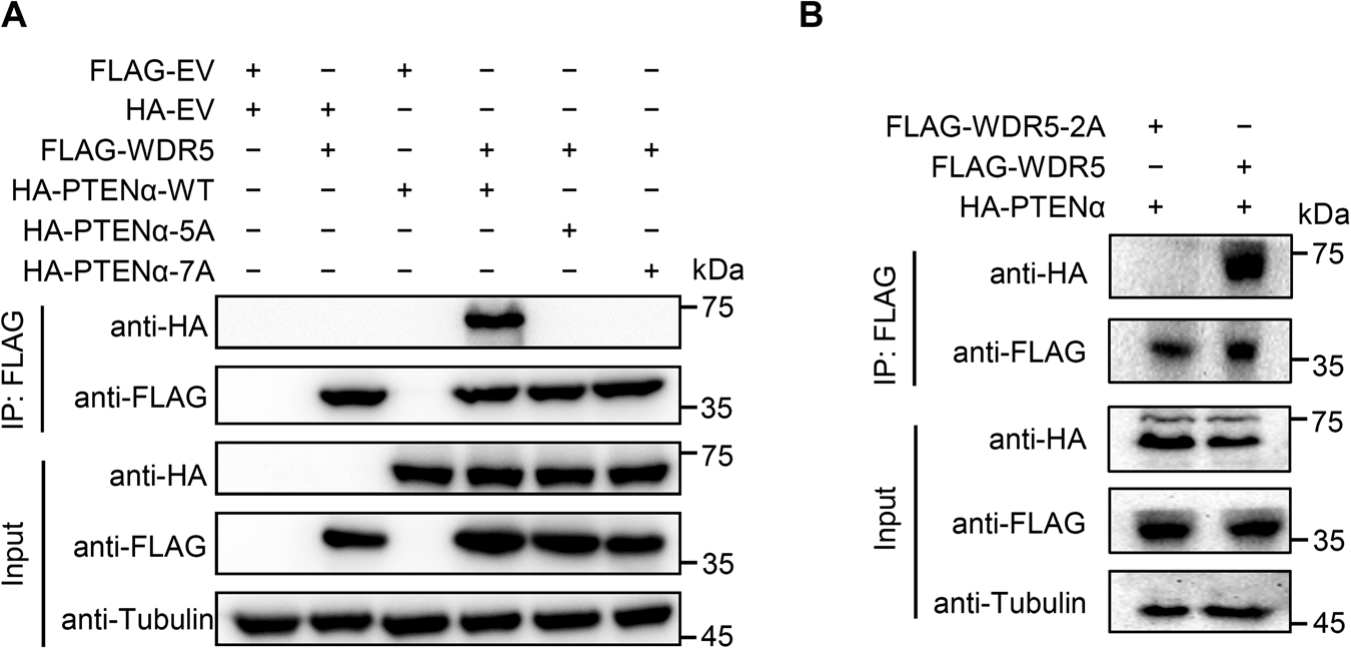
Mutation of the interacting residues affected the interaction between full-length PTENα and WDR5 in cells. **A** Mutation of the key interacting residues of PTENα-NTE affected the interaction with WDR5. **B** Mutation of the key interacting residues of WDR5 affected the interaction with PTENα. HEK293T cells were transfected with the wild-type or F133A/263A (WDR5-2A) mutant 3×FLAG-tagged WDR5 along with the wild-type PTENα, PTENα-5A, or PTENα-7A mutants. Western blotting was performed on the indicated proteins in co-immunoprecipitates (Co-IPs). Full and uncropped Western blots are provided in Supplemental Material.

To confirm that PTENα interacts with WDR5 through its SSSRRSS motif in cells, the 3×FLAG-tagged wild-type WDR5 or its F133A/263A (WDR5-2A) mutant was co-transfected with either the wild-type HA-tagged PTENα or its 5A and 7A mutants into HEK293T cells. Co-IP assays using an anti-FLAG antibody disclosed that only PTENα was successfully co-immunoprecipitated with WDR5, while PTENα-5A and PTENα-7A did not show any interaction with WDR5 (Fig. 5A). Similarly, PTENα was co-immunoprecipitated with the wild-type WDR5 only, but not with the mutant WDR5 containing the F133A/263A mutations (WDR5-2A) (Fig. 5B). These results were consistent with our findings from the complex structure, ITC and in vitro GST pulldown studies, highlighting the critical role of the PTENα-NTE SSSRRSS motif and the WDR5 WIN binding site for the interaction between PTENα and WDR5 in a cellular context.

**Fig. 6:**
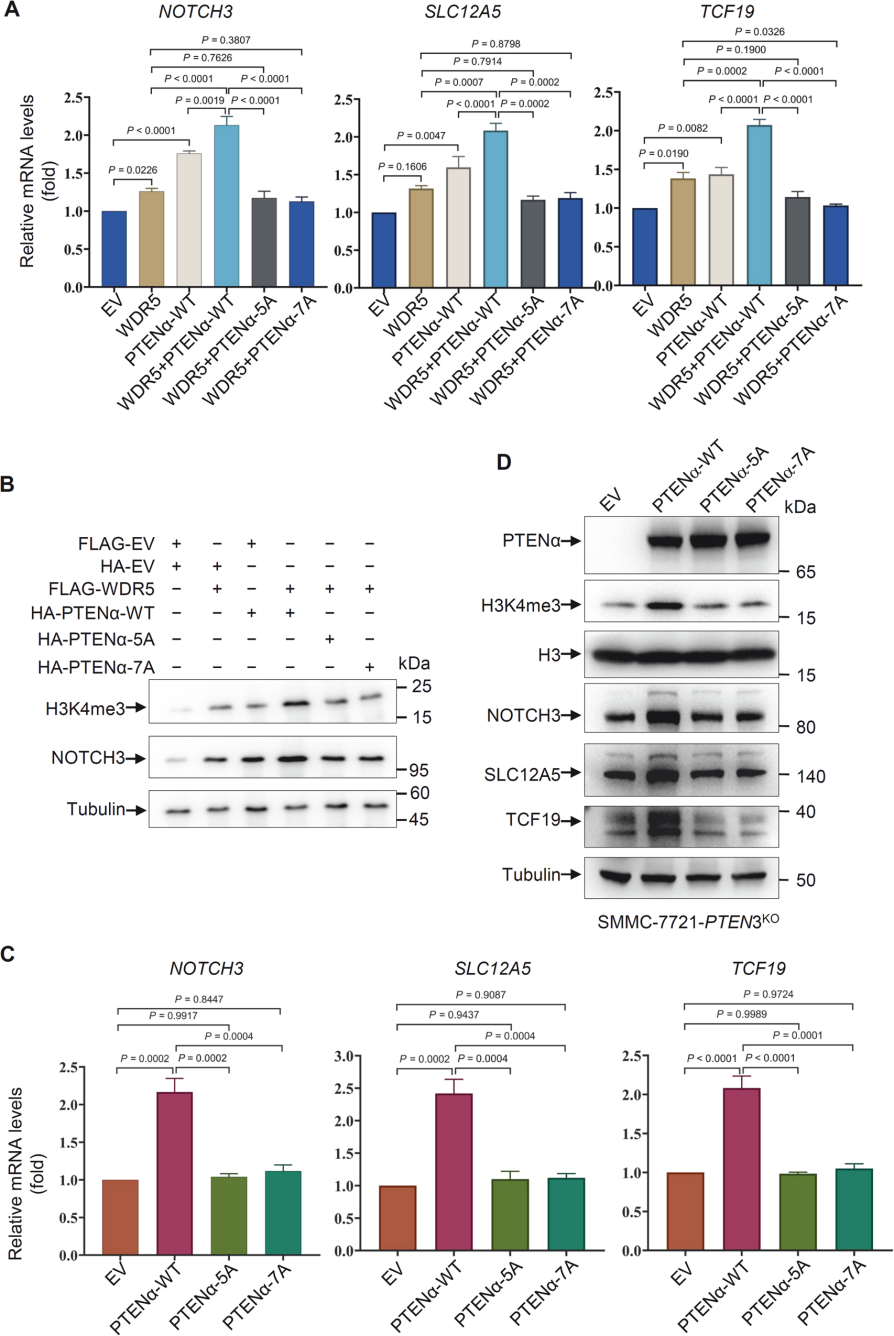
Mutation of the SSSRRSS motif of PTENα-NTE diminishes the transcriptional activity and expression of genes associated with tumorigenicity. **A** Disruption of the interaction between PTENα and WDR5 impaired transcriptional activity of target genes, such as *NOTCH3*, *SLC12A5*, and *TCF19* determined by qRT-PCR in HEK293T cells. **B** Disruption of the interaction between PTENα and WDR5 decreased the protein level of NOTCH3 and trimethylated histone H3K4 determined by Western blotting in HEK293T cells. **C** Disruption of the interaction between PTENα and WDR5 impaired transcriptional activity of target genes, such as *NOTCH3*, *SLC12A5*, and *TCF19* determined by qRT-PCR in *PTEN*3^KO^ SMMC-7721 cells. **D** Disruption of the interaction between PTENα and WDR5 decreased the protein level of NOTCH3, SLC12A5 and TCF19, as well as trimethylated histone H3K4 determined by Western blotting in *PTEN*3^KO^ SMMC-7721 cells. The experiments were repeated three times independently with similar results, and the results of one representative experiment were shown. For (**A**) and (**C**), data represent means ± s.e.m. Statistical significance was determined by two-tailed unpaired *t* test. Pre-stained protein marker: Abclonal, RM19001 (**B**) and ThermoFisher, 26616 (**D**), respectively. Full and uncropped Western blots are provided in Supplemental Material.

The previous study has shown that the interaction between PTENα/β and WDR5 plays a crucial role in recruiting the SET1/MLL methyltransferase complex to regulate the downstream H3K4me3 level and expression of oncogenes such as *NOTCH3*, *SLC12A5*, and *TCF19* [15]. To validate the importance of the SSSRRSS motif of PTENα-NTE in regulating the expression of these oncogenes and the H3K4me3 level, qRT-PCR and Western blot experiments were conducted by using the above HEK293T cells. The results demonstrated that co-expression of WDR5 and wild-type PTENα had a more potent effect compared to the expression of either WDR5 or wild-type PTENα on the mRNA levels of *NOTCH3*, *SLC12A5*, as well as *TCF19*, and the levels of H3K4me3 modification and NOTCH3 (Fig. 6A, B). However, this enhanced effect was disrupted in the mutant of the SSSRRSS motif, which showed comparable effects to that of WDR5 expression alone (Fig. 6A, B). To further confirm the regulatory role of the PTENα-NTE SSSRRSS motif, qRT-PCR and Western blot experiments were performed using a stable *PTEN*3 (PTEN, PTENα, and PTENβ) knockout cell line (SMMC-7721-*PTEN*3^KO^) to prevent residual signal coming from endogenous PTEN and its isoforms. Ectopic expression of wild-type PTENα, but not PTENα-5A/7A, restored the promotion of NOTCH3, SLC12A5, and TCF19 on both mRNA levels and protein levels, as well as the H3K4me3 level in these cells (Fig. 6C, D). Overall, these results indicate that the SSSRRSS motif of PTENα-NTE plays a critical role in interacting with WDR5, as well as modulating downstream epigenetic factors and gene expression related to tumorigenicity.

### Mutation of the SSSRRSS motif of PTENα-NTE stops tumor promotion by PTENα

**Fig. 7:**
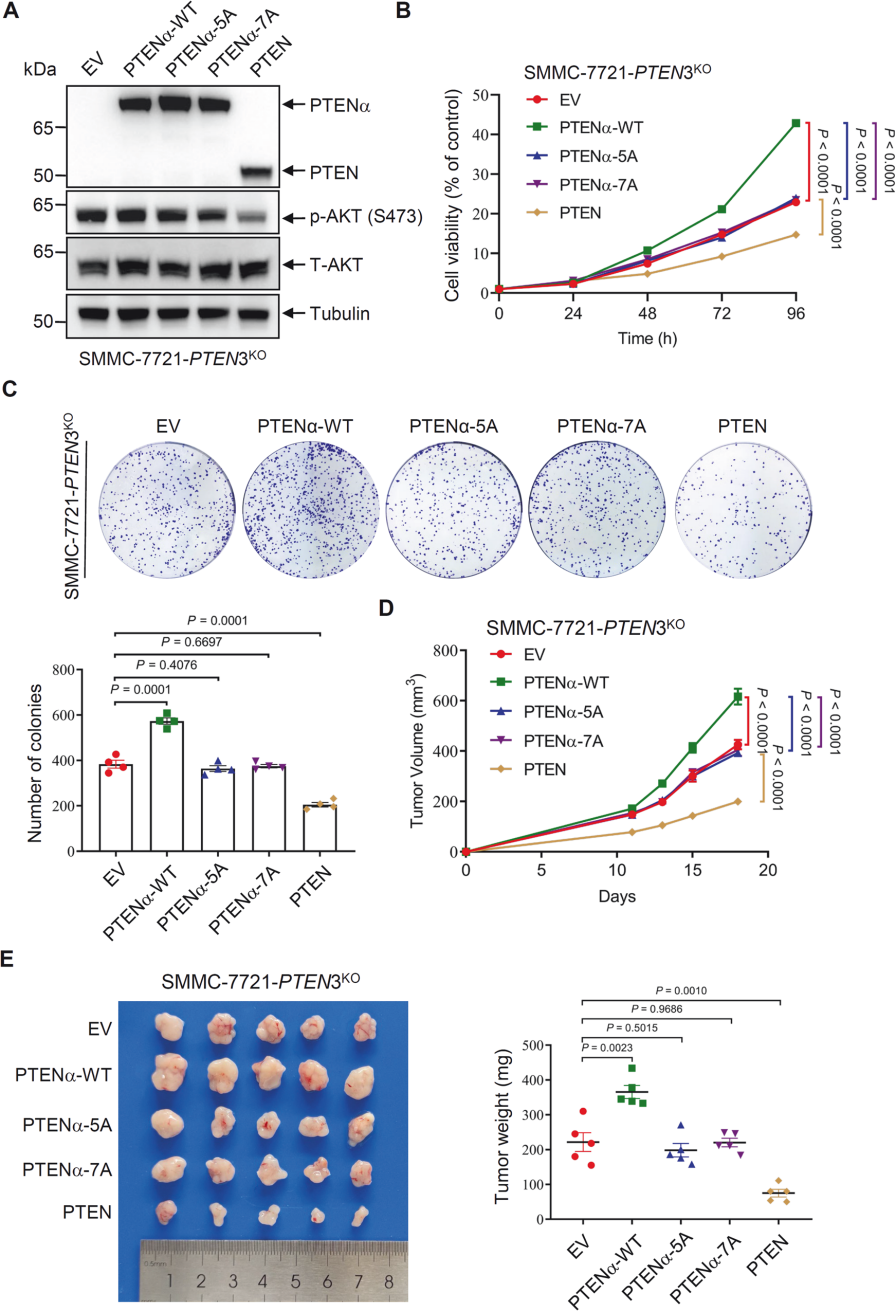
Mutation of the SSSRRSS motif of PTENα-NTE stops tumor promotion by PTENα. **A** The efficiency of rescue of *PTEN*3^KO^ SMMC-7721 cells by ectopic expression of wild-type PTEN, PTENα or its mutants. SMMC-7721 *PTEN*3^KO^ cells were transduced by lentiviruses encoding wild-type PTEN, PTENα or its mutants, followed by Western blotting of the indicated proteins. Pre-stained protein marker: ThermoFisher, 26616. **B**, **C** Disruption of the interaction between PTENα and WDR5 by the key interacting residues point mutation impaired tumor cell growth and tumorigenic capacity. CCK8 assays (**B**) and colony formation assays (**C**) were used to determine the proliferation and tumorigenic capacity of these cells. **D**, **E** Disruption of the interaction between PTENα and WDR5 destroyed the promotion of tumorigenesis by PTENα. The transfected cells were subcutaneously injected into nude mice (1 × 10^6^ cells per mouse; *n* = 5 mice per group). Tumor volumes were measured on different days (**D**). On day 18, tumors were harvested, photographed **(left)**, and weighed (**right**) (**E**). The experiments were repeated three times (twice for animal experiments without blinding) independently with similar results, and the results of one representative experiment were shown. For (**B**–**E**), data represent means ± s.e.m. Statistical significance was determined by two-way ANOVA (**B**, **D**) or two-tailed unpaired *t* test (**C**, **E**). p-AKT phosphorated AKT, T-AKT total AKT. Full and uncropped Western blots are provided in Supplemental Material.

To investigate the role of the SSSRRSS motif of PTENα-NTE in promoting tumorigenesis of SMMC-7221 cancer cells, mutants of PTENα-5A/7A were used to examine whether they could impede PTENα-mediated tumorigenesis promotion in the SMMC-7721-*PTEN*3^KO^ cell line. Ectopic expression of wild-type PTENα, but not PTENα-5A/7A, restored the pro-proliferative effect in these cells, while the ectopic expression of PTEN inhibited cell proliferation, consistent with its function as a tumor suppressor (Fig. 7A–C). Additionally, ectopic expression of PTENα had no impact on AKT activation in *PTEN*3^KO^ SMMC-7721 cells (Fig. 7A, compared with empty vector, EV group, and canonical PTEN group), consistent with the previous study [15]. When these cells were subcutaneously inoculated into nude mice, the ectopic expression of wild-type PTENα accelerated tumorigenesis, while the PTENα-5A/7 A groups did not show significant difference from the control group (Fig. 7D, E).

To further investigate the role of the interaction between WDR5 and PTENα-NTE SSSRRSS motif in cancer progression, a *WDR5* knockout SMMC-7721 cell line (SMMC-7721-*WDR5*^KO^) was generated and rescued it with empty vector (EV), wild-type WDR5, or WDR5-2A mutant (F133A/263A) (Supplementary Fig. S8A). The results revealed that ectopic expression of wild-type WDR5 promoted potent cell proliferation and tumor growth in comparison to all other cell lines (Supplementary Fig. S8B–E). Notably, ectopic expression of the WDR5-2A mutant partially rescued cell proliferation and the tumor-promoting effect owing to the retention of the WBM (WDR5-binding motif) site, which interacts with other oncoproteins such as MYC [23]. Furthermore, in addition to PTENα, WDR5 also interacts with various proteins through its WIN site and these interactions are extensively reported to be involved in tumor progression [40, 41]. Thus, this phenotype cannot be attributed solely to the loss of interaction with PTENα/β alone. Taken together, our findings suggest that the interaction between the SSSRRSS motif of PTENα and WDR5 is indispensable for PTENα-mediated cancer cell proliferation and tumor growth. However, this interaction is not the only requirement for pro-tumor activity of WDR5. Interactions of WDR5 with other proteins such as MYC may also play a role in promoting tumor growth. Overall, these results provide new insights into the mechanisms underlying liver cancer cell proliferation and tumor growth, highlighting potential targets for developing new therapeutic strategies.

## Discussion

In this study, we uncovered the molecular mechanism of how PTENα/β-NTE promotes cancer progression and identified a novel binding motif, SSSRRSS, a common fragment in the PTENα/β-NTE domain that interacts with the WDR5 WIN site. The WDR5 WIN site, one of the two well-known binding sites of WDR5, has been found to interact with various ligands, exerting different physiological functions, many of which are linked to cancer development [36–39]. Typically, this site recognizes its ligands by enveloping the conserved arginine residue of the ligands by its central negatively charged channel and accommodating a small side-chain residue (P_-1_ position) preceding the conserved arginine residue (P_0_ position), such as alanine [20, 21, 50–52] or cysteine [46], via a shallow surface pocket. However, a new -RR- WIN site binding motif was discovered that features a larger side-chain arginine at the P_-1_ position. Structural analysis revealed that a specific intramolecular hydrogen bond of the P_-1_ arginine stabilizes its side chain, significantly reducing steric hindrance to allow for proper interaction with WDR5. To our knowledge, our structure is the first structure of PTENα-NTE since it was reported ten years ago. Furthermore, our findings provide new insights into the structural determinants governing the interaction between PTENα/β-NTE and WDR5, as well as redefine the sequence characteristics of the WDR5 WIN site ligands.

Further structural and binding studies demonstrated that PTENα/β-NTE interacts with the WDR5 WIN site solely through the SSSRRSS WIN motif and the mutations of the key interaction residues disrupt this association significantly. In vitro and in vivo studies revealed that the SSSRRSS fragment is indispensable for the pro-tumor activity of PTENα, while the F133/F263 residues of WDR5, essential for the interaction with PTENα/β and other WIN site ligands, are necessary but not the only requirement for the pro-tumor activity of WDR5. In fact, WDR5 also interacts with MYC, a well-known oncoprotein broadly overexpressed in some cancers, through its WBM site to promote tumorigenesis [23], and other proteins through its WIN site and these interactions are extensively reported to be involved in tumor progression [40, 41]. As PTENα and PTENβ share the same SSSRRSS fragment, our findings regarding PTENα are also applicable to PTENβ. Overall, our studies emphasize the role of the PTENα/β-WDR5 interaction in promoting oncogenic processes and provide a structural basis for development potential therapeutic targets for cancer treatment. Additionally, our studies suggest that the combined use of inhibitors targeting both the WIN site and the WBM site of WDR5 would be more effective in suppressing tumor growth than using the individual site inhibitor, which aligns with a recent research [53].

In addition to their intracellular localization, the PTENα/β proteins can also be released into the extracellular space, where they are cleaved by the Furin proteinase. This cleavage generates a long C-terminal fragment, which has been shown to have significant tumor-suppressive properties [14]. Unfortunately, in liver cancer tissues, the expression of Furin is low and the cleavage of PTENα/β is reduced. Therefore, a potential therapeutic strategy for liver cancer could involve combining a PTENα/β-WDR5 protein-protein interface inhibitor with an activator of Furin. Further research is needed to test this hypothesis.

In conclusion, our study has revealed a novel binding motif within PTENα/β-NTE interacting with the WIN site of WDR5 to control downstream histone methylation and increase tumorigenic genes expression, cell proliferation, and tumor growth. Disrupting the PTENα/β-WDR5 interaction by point mutations of the key interacting residues attenuates these effects, which indicates that novel therapies for cancer treatment could be potentially developed by targeting the interaction between PTENα/β and WDR5. Additionally, inhibiting both the WIN and WBM sites of WDR5 may further improve therapeutic efficacy. Overall, our research advances our understanding of the molecular mechanisms underlying cancer biology and offers promising possibilities for therapeutic interventions.

## Materials And Methods

### Plasmids, cell line and antibodies

The *E. coli* expression vectors, pET28GST-LIC, pET28a-MHL and pET32a-LIC were constructed in our laboratory. All the primers used in this study were provided in Supplementary Table S1.

Antibodies used in this study are as follows: mouse monoclonal anti-β-tubulin antibody (Sigma-Aldrich, Cat# T4026, 1:5000), mouse monoclonal anti-FLAG M2 antibody (Sigma-Aldrich, Cat# A8592, 1:5000), rabbit monoclonal anti-HA antibody (Sigma-Aldrich, Cat# H6908, 1:1000), rabbit monoclonal anti-NOTCH3 antibody (Cell Signaling Technology, Cat# 5276, 1:1000), rabbit monoclonal anti-H3K4me3 antibody (Cell Signaling Technology, Cat# 9751, 1:1000), rabbit monoclonal anti-PTEN antibody (Cell Signaling Technology, Cat# 9559, 1:1000), rabbit monoclonal anti-AKT antibody (Cell Signaling Technology, Cat# 4691, 1:1000), rabbit monoclonal anti-p- AKT (S473) antibody (Cell Signaling Technology, Cat# 4060, 1:1000), rabbit monoclonal anti-SLC12A5 antibody (Proteintech, Cat# 28724-1-AP, 1:1000), rabbit monoclonal anti-TCF19 antibody (Affinity Bioscience, Cat# DF9971, 1:1000).

### Cell lines and cell culture

Human embryonic kidney 293 T (HEK293T) cells and human liver cancer SMMC-7721 cells were purchased from ATCC and Cell Bank of the Chinese Academy of Sciences in Shanghai, respectively. All the cell lines used in this study were cultured in DMEM supplemented with 10% fetal bovine serum, and underwent authentication using the short tandem repeat (STR) profile method and tested negative for mycoplasma contamination by PCR.

### Protein expression and purification

The DNA fragments of WDR5 (residues 22–334 or 1–334) and PTENα-NTE (residues 1–173) were subcloned into a pET28a-MHL vector to generate N-terminal 6×His-TEV-tagged fusion protein. Additionally, WDR5 (residues 22–334) was subcloned into a modified pET28GST-LIC vector to generate N-terminal GST-6×His-tagged fusion protein. PTENα (residues 1–576) was subcloned into a pET32a-LIC vector to generate N-terminal Trax-6×His-S-TEV-tagged fusion protein. All the plasmids were constructed using seamless assembly cloning (ABclonal Technology, RK21020) and confirmed by sequencing (Azenta Life Sciences).

The recombinant proteins were overexpressed in *E. coli* BL21 (DE3) Codon plus RIL (Stratagene, 230280) at 15 °C for 24 h under induction with 0.25 mM IPTG (isopropyl-β-D-thiogalactoside) at an OD_600_ value of 0.8. They were then purified using affinity chromatography on Ni-nitrilotriacetate resin (GE Healthcare, 17526802) followed by TEV protease treatment to remove the tag for ITC assays and crystallization. The buffer conditions for Ni-affinity chromatography were as follows: lysis buffer: 20 mM Tris-HCl, pH 7.5, 250 mM NaCl, 5% glycerol, and 5 mM β-mercaptoethanol; wash buffer: 20 mM Tris-HCl, pH 7.5, 1 M NaCl, and 40 mM imidazole; elution buffer: 20 mM Tris-HCl, pH 7.5, 250 mM NaCl, and 250 mM imidazole.

WDR5 (residues 22–334 or 1–334) and PTENα (residues 1–576) proteins were further purified using a Superdex200 gel-filtration column (GE Healthcare, 28989335) with a buffer containing 20 mM Tris-HCl, pH 7.5, 150 mM NaCl, and 1 mM DTT. PTENα-NTE (residues 1–173) was further purified by ion exchange chromatography (HiTrap SP HP column, GE Healthcare, 17115201) using buffers with 20 mM Tris-HCl, pH 7.5, 1 mM DTT, adding either 50 mM NaCl (low salt buffer) or 1 M NaCl (high salt buffer), followed by dialysis using a buffer containing 20 mM Tris-HCl, pH 7.5, and 150 mM NaCl (ITC buffer). All the mutations involved in this study were constructed using Fast Mutagenesis System Kit (Transgene, FM111-02) according to the manufacturer’s instruction and confirmed by DNA sequencing. Mutants were overexpressed and purified as the wild-type constructs described above. All the proteins were concentrated using Amicon Ultra-15 Centrifugal Filter Units (Millipore Corporation, UFC901024).

### Isothermal titration calorimetry (ITC)

For the ITC measurement, concentrated proteins were diluted into the ITC buffer, while lyophilized peptides (Shanghai Apeptide CO., Ltd or GLS GL Biochem (Shanghai) Ltd.) were dissolved in the same buffer, and the pH was adjusted by adding 2 M NaOH dropwise. Peptide concentrations were estimated based on their mass. All measurements were conducted in duplicate at 25 °C, utilizing an iTC-200 (MicroCal, Inc.) microcalorimeter.

In the cell chamber, a protein with a concentration of 50 µM was placed, and peptides or proteins with a concentration of 750 µM in the syringe were injected into the cell chamber for 20 successive injections with a spacing of 150 seconds. Control experiments were carried out under identical conditions to determine the heat signals that resulted from injecting peptides or proteins into the buffer. Data were fitted using the single-site binding model within the Origin software package (MicroCal, Inc.).

### Protein crystallization

For the crystals of WDR5^22–334^-PTENα-NTE^1–173^, purified WDR5^22–334^ was mixed with PTENα-NTE^1–173^ at a molar ratio of 1:1 and with trypsin at a mass ratio of 1:1000 (trypsin: protein mixture) [54, 55]. Then, the mixture was crystallized using the sitting-drop vapor diffusion method at 18 °C by adding 0.5 μL of the protein mixture (8 mg/mL) with 0.5 μL of the reservoir solution. The complex of WDR5^22–334^-PTENα-NTE^1–173^ crystallized in a buffer containing 0.2 M lithium sulfate monohydrate, 0.1 M HEPES, pH 7.5, and 25% w/v polyethylene glycol 3,350. As for the crystals of WDR5^1–334^, purified WDR5^1–334^ protein was mixed with PTENα-NTE^116–148^ peptide at a molar ratio of 1:3, and crystallized in a buffer containing 0.1 M magnesium formate dihydrate and 15% w/v polyethylene glycol 3,350. Before flash-freezing crystals in liquid nitrogen, crystals were soaked in a cryoprotectant consisting of 85% reservoir solution and 15% glycerol.

### Data collection and structure determination

The diffraction data of the crystals were collected at beamline BL18U1 of the Shanghai Synchrotron Radiation Facility (SSRF) at 100 K. Diffraction images were processed using XDS [56]/POINTLESS [57]/AIMLESS [58]. PHASER [59] software was used for molecular replacement searches. COOT [60] was used for interactive model building. Following initial restrained model refinement with REFMAC [61], the model was automatically rebuilt with ARP/wARP [62]. Subsequent model refinement was carried out using REFMAC and PHENIX [63]. The crystal structure of WDR5 (PDB entry: 2H9M) was used as a coordinate for molecular replacement. REFMAC was applied for restrained model refinement [61]. Crystal diffraction data and refinement statistics for the structures are presented in Table 1. All the structural figures were generated using PyMOL.

### GST pulldown assay

The purified wild-type and F133A/263 A (WDR5-2A) mutant GST-tagged fusion protein WDR5^22–334^ (100 μg) was bound to Glutathione Sepharose 4B (GE Healthcare, 28952360) for 1 h at 4 °C. After washing with a buffer containing 20 mM Tris-HCl, pH 7.5, 150 mM NaCl, and 0.1% Triton X-100 for three times, the bound GST-tagged fusion proteins were incubated with purified tag-removed recombinant PTENα-NTE^1–173^ or full-length PTENα^1–576^, along with their respective mutants (300 μg). This incubation was carried out overnight at 4 °C. Following another round of washing with the same buffer, the pulldown samples were eluted by adding 1× SDS protein loading buffer and loaded onto SDS-polyacrylamide gels and analyzed by Coomassie brilliant blue (CBB) staining as previously described [64].

### Co-IP and Western blotting

Eukaryotic expression plasmids, including the wild-type and F133A/263 A (WDR5-2A) mutant 3×FLAG-WDR5, HA-PTENα, HA-PTENα-5A, and HA-PTENα-7A, were individually or co-transfected into HEK293T cells using Lipofectamine 2000 (ThermoFisher Scientific, 11668027) following the manufacturer’s protocol. The cells were harvested and lysed using IP (immunoprecipitation) lysis buffer containing 50 mM HEPES, pH 7.5, 150 mM NaCl, 10% glycerol, 1% Triton X-100, 1.5 mM MgCl_2_, and 1× protease inhibitor mixture (MCE, 180528) 48 h post-transfection as a previously described method with minor modifications [65, 66]. Briefly, the lysates were sonicated with five cycles of 0.3 s/0.7 s per mL and incubated for 30 min at 4 °C and centrifuged at 12,000 rpm for 10 min at 4 °C. The resulting supernatant were incubated with 20 μL of Anti-FLAG-Affinity-Gel (Selleck.cn, B23101) overnight at 4 °C, followed by three washes with wash buffer (ITC buffer + 0.5% Triton X-100), each for 10 min. Then, 50 μL of 1× SDS protein loading buffer was added, and the mixture was boiled for 10 min. Subsequently, it was centrifuged at 12,000 rpm for 10 min, and the supernatant was removed for further analysis.

Input and IP protein samples were separated on a 4–12% Bis-Tris protein gel (GenScript, M41215C) using Tris-MOPS running buffer and then transferred onto a PVDF membrane and blocked overnight in 3% BSA in PBST buffer (PBS + 0.1% Tween 20). The membrane was then incubated with primary antibodies targeting the intended proteins for 1 h, followed by three 10-min washes in PBST. This process was repeated with secondary antibodies. Finally, the membrane was visualized using an Odyssey® CLx Imaging System (LI-COR).

### Quantitative real-time polymerase chain reaction (qRT-PCR)

Total RNA from various cell samples was extracted using TRIzol Reagent (Vazyme, R401-01). Subsequently, 1 μg of RNA was transcribed into complementary DNA (cDNA) using the ABScript III RT-PCR kit (ABclonal Technology, RK20429). The qPCR was performed using an SYBR Green reaction mix (ABclonal Technology, RK21203) with a LightCycler 96 System (Roche, Basel, Switzerland). Relative expression levels of target genes were calculated utilizing the 2^-ΔΔCt^ method (Ct, cycle threshold).

### Lentivirus-mediated *WDR5*/*PTEN*3 knockout

To generate *PTEN* or *WDR5* knockout in SMMC-7721 cell line, the following protocol was utilized: Cells were transfected with LentiCRISPR v2 plasmids containing specific sgRNAs: *PTEN* targeting sequence (sgRNA: ACAAAAGGAGATATCAAGAGG) or *WDR5* targeting sequence (sgRNA: TCTGAGTGGCGGATGACGAA). Subsequently, cells were subjected to puromycin selection. After selection, cells were diluted, and individual colonies were isolated. The sgRNA knockout efficacy was assessed by Western blot analysis. PCR and DNA sequencing were used to confirm homozygous gene locus editing.

### Lentiviral transduction

Lentivirus was generated by co-transfecting HEK293T cells with the lentiviral construct pCMV-dR8.91 (Δ8.9) plasmid and the pMDG envelope-expressing plasmid using X-tremeGENE 9 DNA Transfection Reagent (Roche, 6365779001). Viral supernatant was collected within 24–48 h post-transfection for subsequent infection of the target cells.

### CCK-8 and colony formation assays

For CCK-8 assays, 2 × 10^3^ SMMC-7721 cells from different groups were seeded into each well of a 96-well plate. Cell cultures were established at 0 h (3 h after cells seeded was denoted as 0 h), 24 h, 48 h, 72 h, and 96 h. At each time point, 10 μL of CCK8 reagent (Selleck.cn, B34302) was added to each well. After 3 h of incubation, the optical density (OD) value was measured at 450 nm. Colony formation assays were performed by adding a total of 1000 SMMC-7721 cells to each group. After 10 days of culture, the colonies were fixed, stained, and counted by ImageJ.

### Mouse studies

A total of 1×10^6^ cells suspended in 100 μL of serum-free media were subcutaneously implanted into female nude mice aged 4 to 6 weeks (*n* = 5 mice per group, repeated twice). Tumor volumes were regularly monitored using calipers and calculated using the formula: length × (width)^2^/2. In accordance with animal care and ethical guidelines, the largest subcutaneous tumor mass on one flank was maintained below 1 cm^3^. All animal care and experimental procedures were conducted in strict compliance with ethical regulations governing animal research and were approved by the committee for the humane treatment of animals at Shanghai Jiao Tong University School of Medicine.

### Statistics and reproducibility

The statistical analyses were described in the figure legends. The tests used included two-tailed unpaired Student’s *t* test, two-way analysis of variance (ANOVA), using Microsoft Excel and GraphPad Prism 7 (GraphPad Software). Data were represented as means ± s.e.m. The experiments were repeated independently 2–3 times with similar results, as indicated in the figure legends.

### Supplementary information

Supplemental Material

## Data Availability

The coordinates and structure factors of this study were deposited in the Protein Data Bank (PDB) with accession codes 8X3S and 8X3R for the complex of WDR5-PTENα-NTE and WDR5 ligand-free structure, respectively. All other relevant data supporting the key findings of this study were available within the article and its supplementary information file or from the corresponding authors upon reasonable request.
