# Supplementary material for: The NTE domain of PTENα/β promotes cancer progression by interacting with WDR5 via its SSSRRSS motif
Source: Cell Death Dis. 2024 May 14;15(5):335. doi: 10.1038/s41419-024-06714-6 (PMC11094138; doi:10.1038/s41419-024-06714-6)
Supplement: Supplementary file 1 — Supplemental Material [file 41419_2024_6714_MOESM1_ESM.pdf]

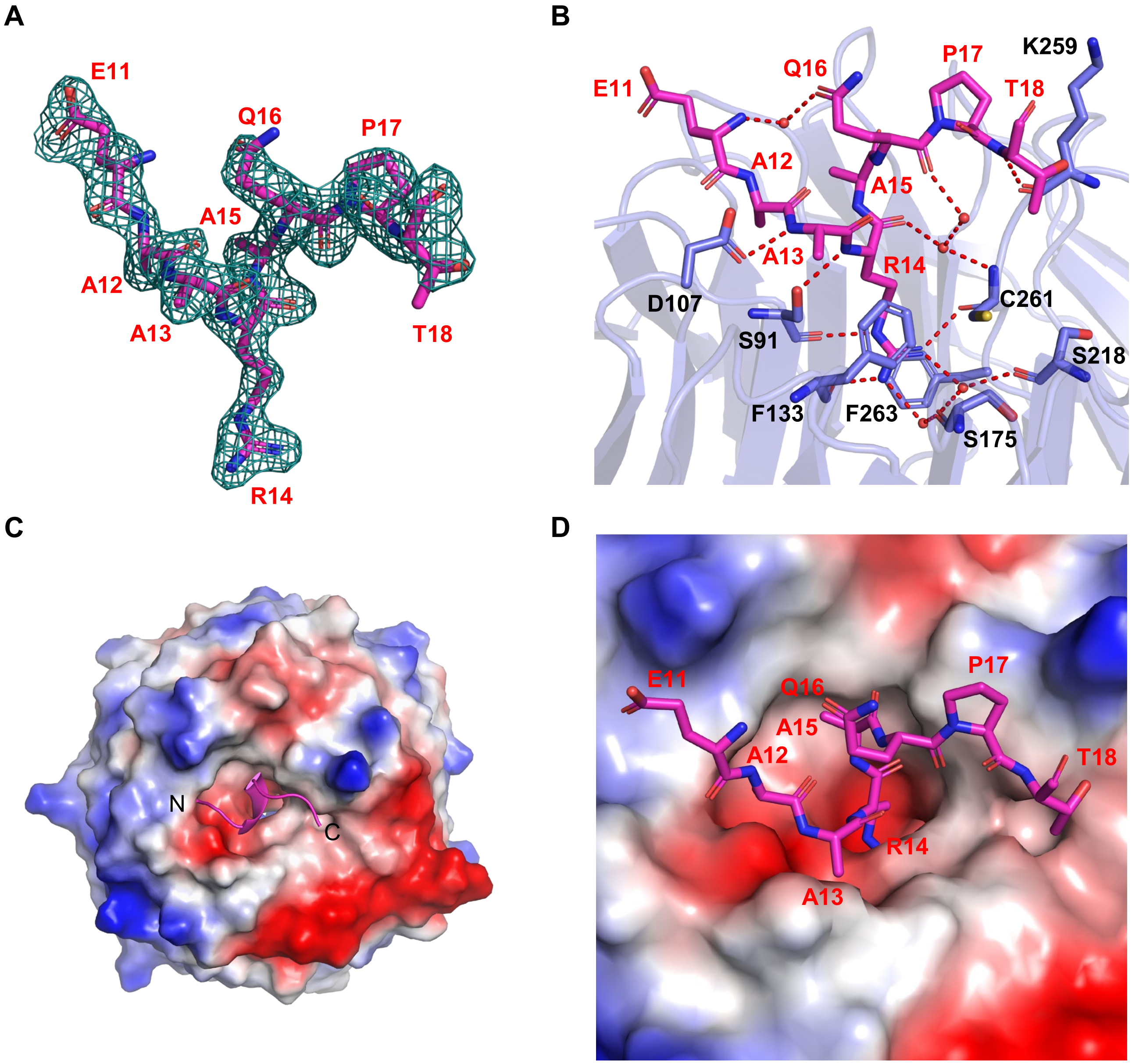

Fig. S1 Crystal structure of WDR5$^{1\text{-}334}$ indicates WDR5$^{32\text{-}334}$ in complex with its N-terminus, WDR5$^{11\text{-}18}$. (A) Fo-Fc omit map of WDR5$^{11\text{-}18}$ contoured at 1σ level. **(B)** Interaction between WDR5$^{32\text{-}334}$ and WDR5$^{11\text{-}18}$. Amino acid residues of WDR5 involved in the interaction were shown as sticks. Key hydrogen bonds were depicted as red dash lines and key water molecules were indicated as red sphere. **(C, D)** Electrostatic potential surface view of WDR5$^{32\text{-}334}$ in complex with WDR5$^{11\text{-}18}$. The WDR5$^{11\text{-}18}$ was shown as cartoon **(C)** and sticks **(D)**, respectively.

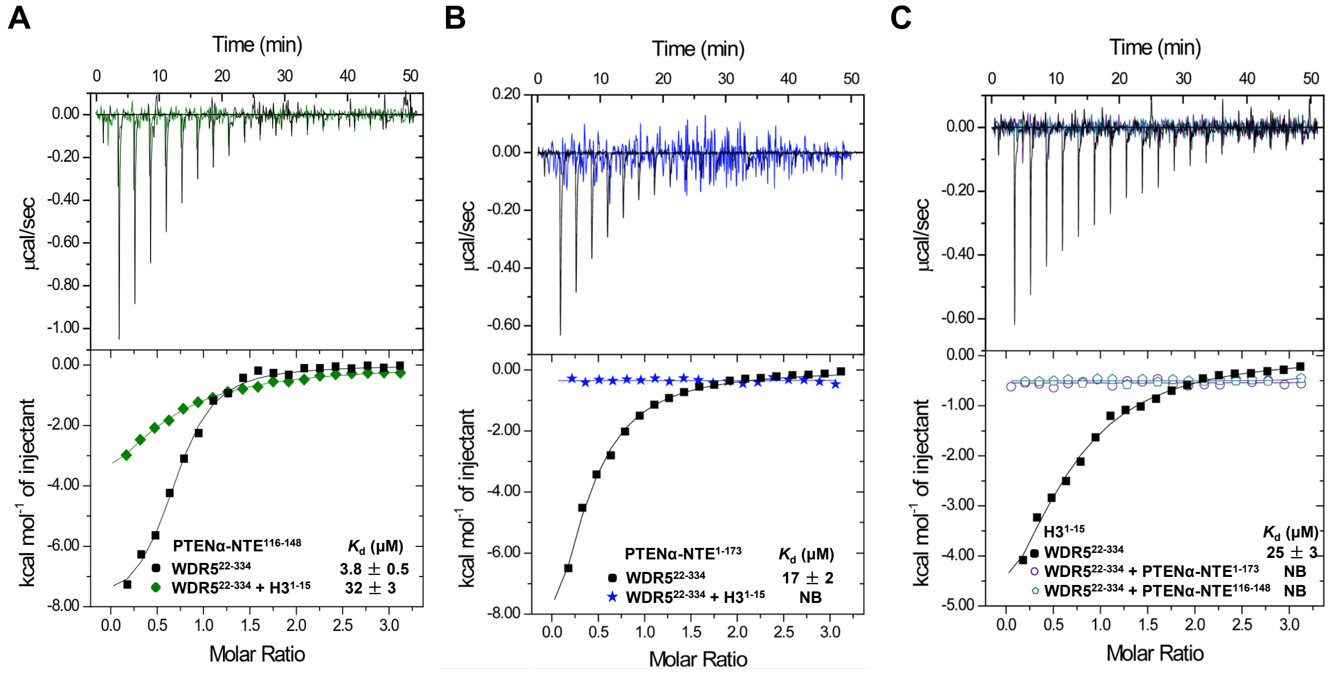

Fig. S2 PTENα-NTE interacts with WDR5 exclusively through the WIN binding site. **(A)** Pre-incubation of WDR5 with unmodified histone H3 peptide weakened the binding of PTENα-NTE$^{116-148}$ to WDR5. ITC binding curves for the titration of PTENα-NTE$^{116-148}$ to WDR5 alone or a mixture of WDR5 with unmodified histone H3 peptide at a molar ratio of 1:1 using an iTC-200 microcalorimeter (MicroCal, Inc.). **(B)** Pre-incubation of WDR5 with unmodified histone H3 peptide destroyed the binding of PTENα-NTE$^{1-173}$ to WDR5. ITC binding curves for the titration of PTENα-NTE$^{1-173}$ to WDR5 alone or a mixture of WDR5 with unmodified histone H3 peptide at a molar ratio of 1:1 using an iTC-200 microcalorimeter (MicroCal, Inc.). **(C)** Pre-incubation of WDR5 with PTENα-NTE inhibited the binding of histone H3 peptide to WDR5. ITC binding curves for the titration of unmodified histone H3 peptide to WDR5 alone or a mixture of WDR5 with PTENα-NTE$^{116-148}$ or PTENα-NTE$^{1-173}$ at a molar ratio of 1:1 using an iTC-200 microcalorimeter (MicroCal, Inc.). K_d: dissociation constants (μM); NB: no detectable binding. Related to Fig. 3A-B.

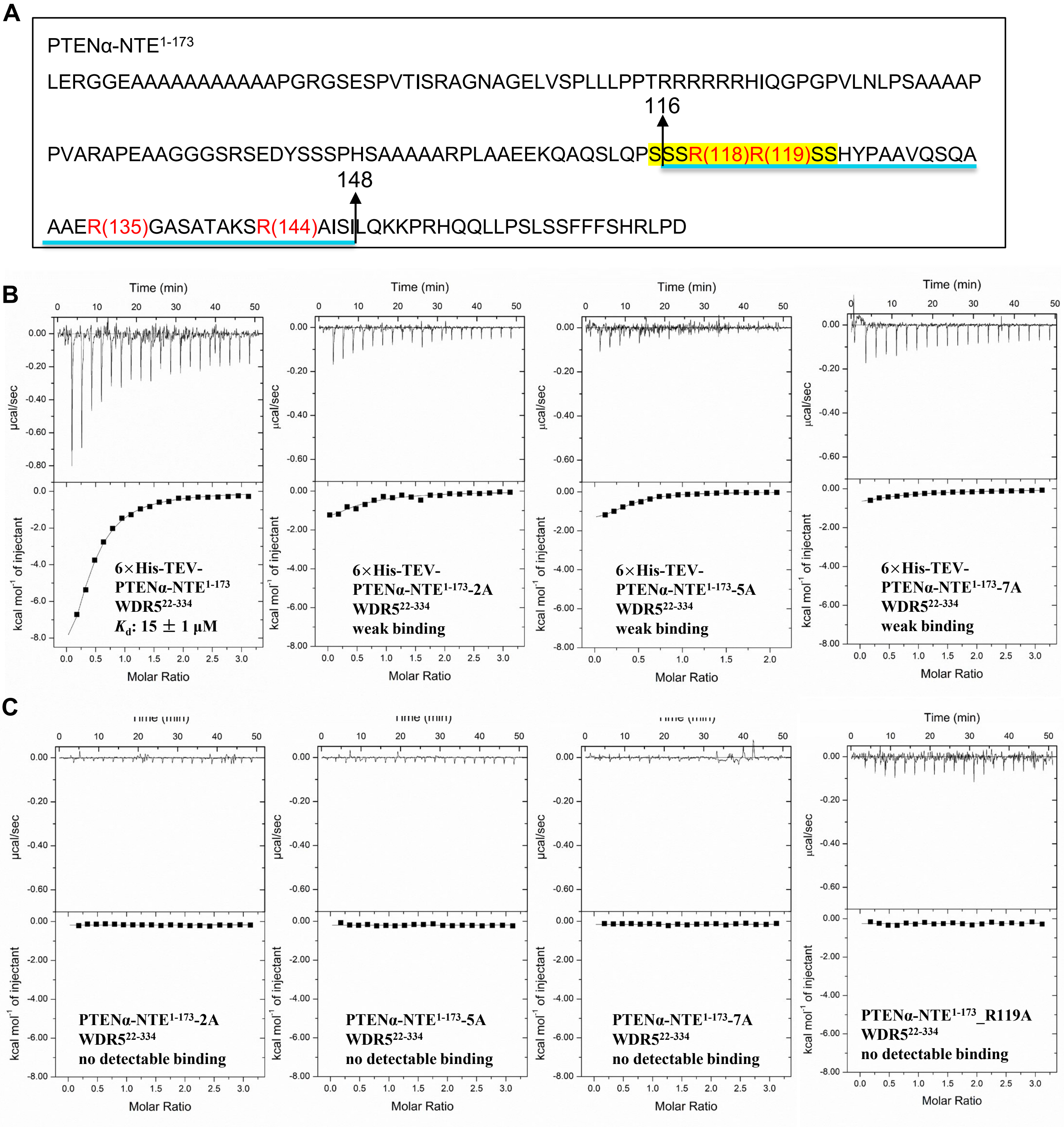

Fig. S3 The 119th arginine of PTENα is critical for the interaction between PTENα-NTE and WDR5. **(A)** There are four arginine residues in the PTENα-NTE$^{116\text{-}148}$ fragment (highlighted in red). **(B)** ITC binding curves for the titration of different mutants of 6×His-TEV-tag fused PTENα-NTE$^{1\text{-}173}$ to WDR5. **(C)** ITC binding curves for the titration of different mutants of 6×His-TEV-tag removed PTENα-NTE$^{1\text{-}173}$ to WDR5. K_d: dissociation constants (μM); 2A: PTENα-NTE_R118A/R119A; 5A: PTENα-NTE_115-119-5A; 7A: PTENα-NTE_115-119-5A_R135A/R144A; 6×His-TEV: MHHHHHHSSGRENLYFQG. Related to Fig. 3D.

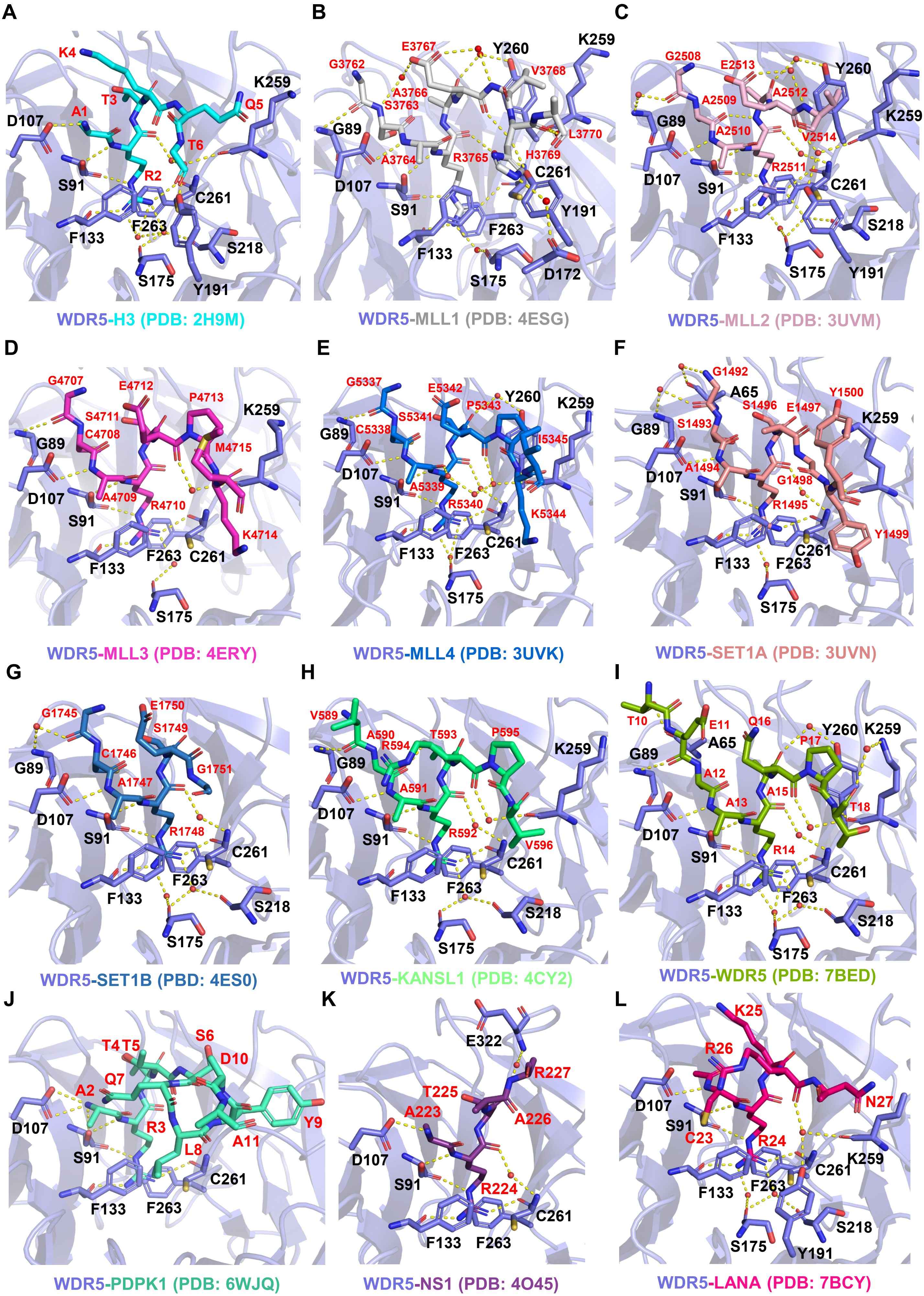
A
K4
T3
A1
D107
Q5
K259
T6
R2
S91
C261
F133
F263
S218
S175
Y191
WDR5-H3 (PDB: 2H9M)
B
E3767
Y260
K259
G3762
V3768
A3766
S3763
G89
L3770
A3764
R3765
H3769
D107
C261
S91
Y191
F263
F133
S175
D172
WDR5-MLL1 (PDB: 4ESG)
C
G2508
E2513
Y260
A2512
A2509
K259
G89
A2510
V2514
D107
R2511
S91
C261
S218
F133
S175
Y191
WDR5-MLL2 (PDB: 3UVM)
D
G4707
E4712
P4713
K259
S4711
C4708
G89
M4715
D107
A4709
R4710
S91
K4714
F263
C261
F133
S175
WDR5-MLL3 (PDB: 4ERY)
E
G5337
E5342
Y260
K259
P5343
S5341
G89
C5338
I5345
D107
A5339
S91
R5340
K5344
C261
F133
F263
S175
WDR5-MLL4 (PDB: 3UVK)
F
G1492
A65
Y1500
S1496
E1497
K259
G89
S1493
D107
A1494
G1498
S91
R1495
Y1499
C261
F133
F263
S175
WDR5-SET1A (PDB: 3UVN)
G
G1745
E1750
S1749
G89
C1746
G1751
A1747
D107
S91
R1748
C261
F263
F133
S218
S175
WDR5-SET1B (PBD: 4ES0)
H
V589
A590
T593
P595
R594
K259
G89
A591
D107
S91
R592
V596
C261
F133
F263
S218
S175
WDR5-KANSL1 (PDB: 4CY2)
I
T10
E11
Q16
Y260
K259
A65
P17
G89
A12
A15
A13
T18
D107
S91
R14
C261
F133
F263
S218
S175
WDR5-WDR5 (PDB: 7BED)
J
T4
T5
S6
D10
Q7
A2
D107
R3
Y9
A11
L8
S91
C261
F133
F263
WDR5-PDPK1 (PDB: 6WJQ)
K
E322
R227
T225
A223
A226
D107
S91
R224
C261
F133
F263
WDR5-NS1 (PDB: 4O45)
L
K25
R26
N27
D107
S91
C23
R24
K259
C261
F133
F263
S218
S175
Y191
WDR5-LANA (PDB: 7BCY)

Fig. S4 Different WIN motif ligand peptides bind to WDR5 using the same arginine-binding pocket. Detail interactions between WDR5 and WIN site ligands. WDR5 was shown in cartoon and colored in slate, WIN site ligands were shown in sticks and colored as follows: unmodified histone H3 (cyan, PDB: 2H9M, **A**); MLL1 (gray, PDB: 4ESG, **B**); MLL2 (pink, PDB: 3UVM, **C**); MLL3 (magenta, PDB: 4ERY, **D**); MLL4 (marine, PDB: 3UVK, **E**); SET1A (salmon, PDB: 3UVN, **F**); SET1B (sky blue, PDB: 4ES0, **G**); KANSL1 (green , PDB: 4CY2, **H**); WDR5 (split pea, PDB: 7BED, **I**); PDPK1 (green cyan, PDB:6WJQ, **J**); NS1 (violet purple, PDB: 4O45, non-structural protein 1 of influenza A H3N2, **K**); LANA (hot pink, PDB: 7BCY, latency-associated nuclear antigen of Kaposi's sarcoma herpesvirus**,** **L**). Related to Fig. 2D and Fig. 4.

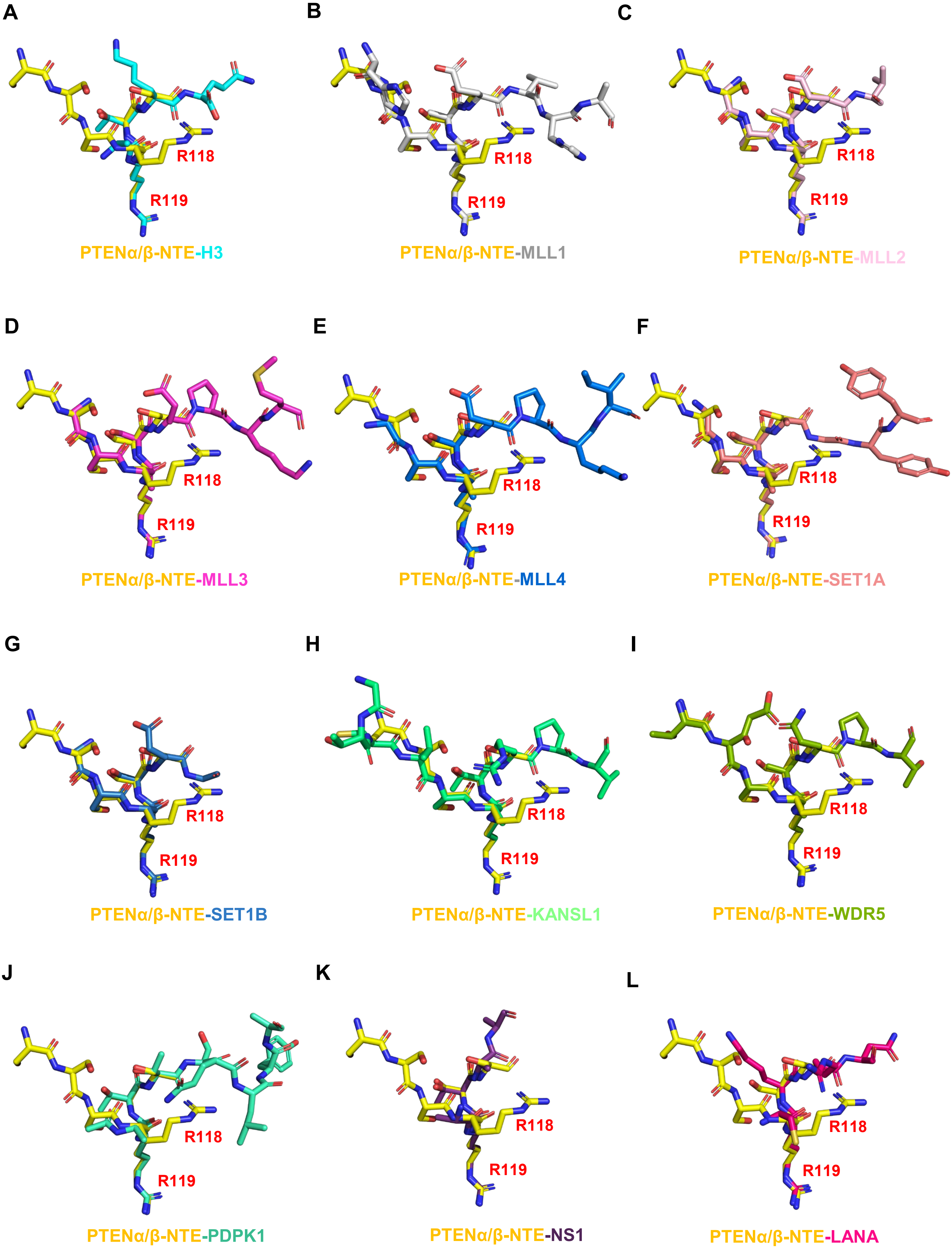
A
R118
R119
PTENα/β-NTE-H3
B
R118
R119
PTENα/β-NTE-MLL1
C
R118
R119
PTENα/β-NTE-MLL2
D
R118
R119
PTENα/β-NTE-MLL3
E
R118
R119
PTENα/β-NTE-MLL4
F
R118
R119
PTENα/β-NTE-SET1A
G
R118
R119
PTENα/β-NTE-SET1B
H
R118
R119
PTENα/β-NTE-KANSL1
I
R118
R119
PTENα/β-NTE-WDR5
J
R118
R119
PTENα/β-NTE-PDPK1
K
R118
R119
PTENα/β-NTE-NS1
L
R118
R119
PTENα/β-NTE-LANA

Fig. S5 The PTENα/β-NTE bind to the WIN site of WDR5. PTENα/β-NTE interact with WDR5 in a manner similar to other WIN motifs. The figures shown superimposition of the PTENα-NTE$^{115\text{-}121}$ (yellow, this work) with published WIN motif from co-crystal structures: unmodified histone H3 (cyan, PDB: 2H9M, **A**); MLL1 (gray, PDB: 4ESG, **B**); MLL2 (pink, PDB: 3UVM, **C**); MLL3 (magenta, PDB: 4ERY, **D**); MLL4 (marine, PDB: 3UVK, **E**); SET1A (salmon, PDB: 3UVN, **F**); SET1B (sky blue, PDB: 4ES0, **G**); KANSL1 (green , PDB: 4CY2, **H**); WDR5 (split pea, PDB: 7BED, **I**); PDPK1 (green cyan, PDB:6WJQ, **J**); NS1 (violet purple, PDB: 4O45, non-structural protein 1 of influenza A H3N2, **K**); LANA (hot pink, PDB: 7BCY, latency-associated nuclear antigen of Kaposi's sarcoma herpesvirus**,** **L**). Related to Fig. 2D and Fig. 4.

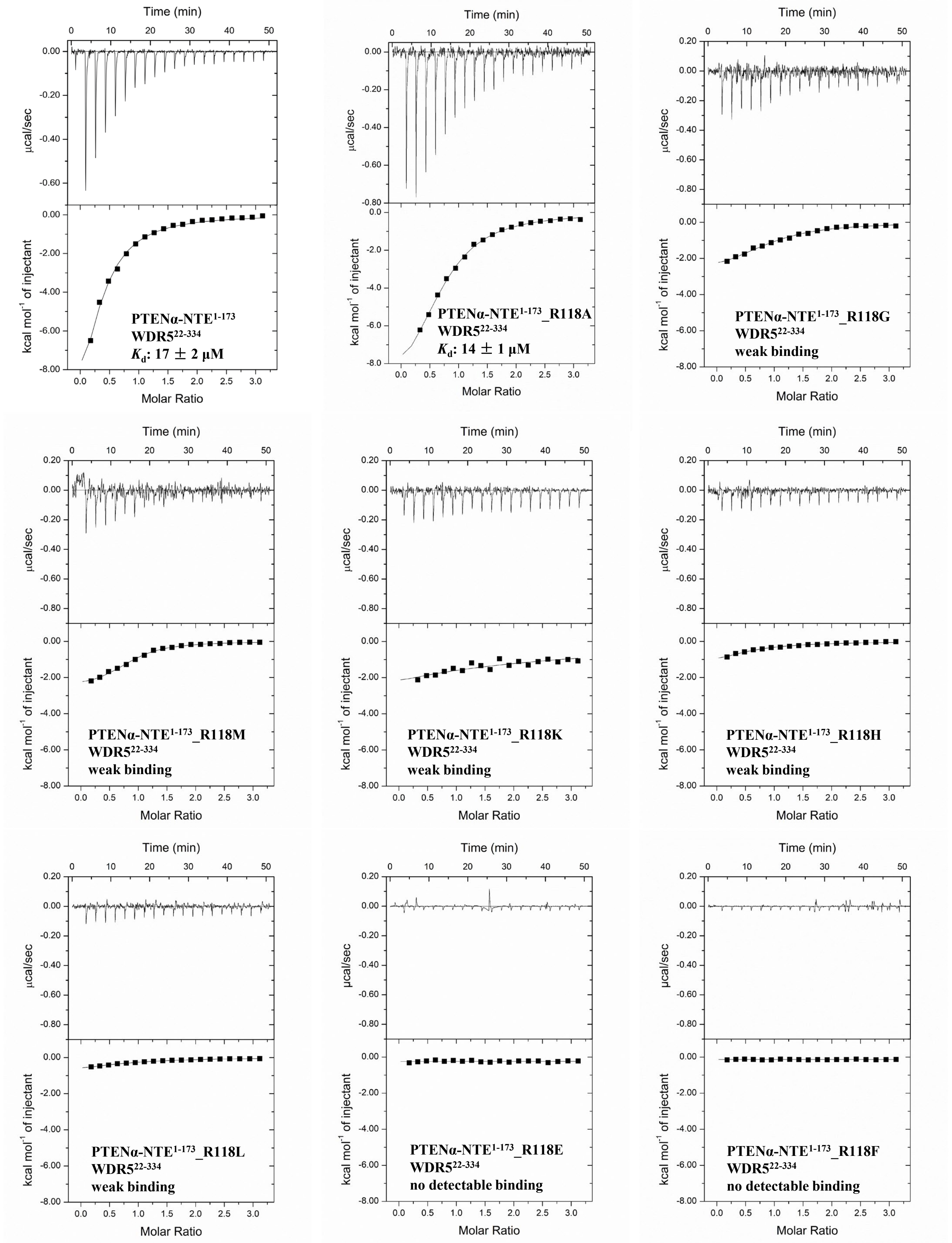

Fig. S6 ITC binding curves for the titration of different R118 mutants of PTENα-NTE$^{1-173}$ to WDR5. K_d: dissociation constants (μM). Related to Fig. 4D.

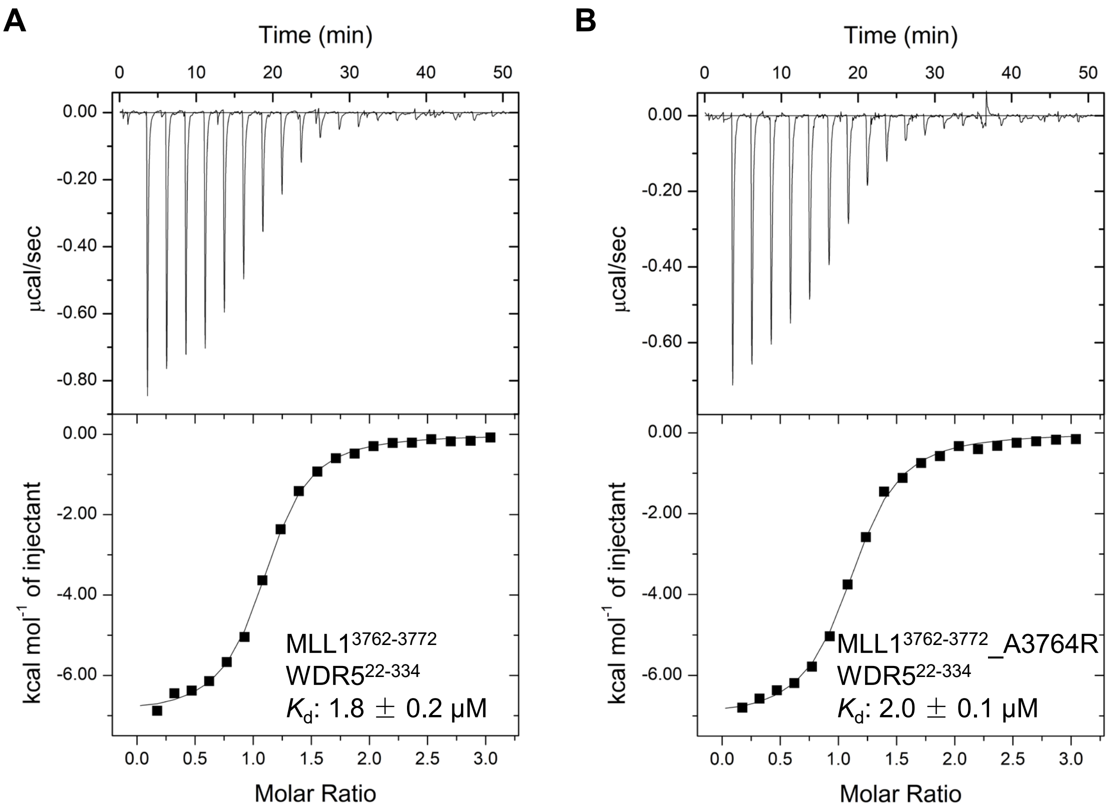

Fig. S7 The arginine at P_{-1} position is acceptable for MLL1 WIN motif, another well-known ligand of the WDR5 WIN site. (A) ITC binding curve for the titration of wild-type $MLL1^{3762\text{-}3772}$ WIN motif to WDR5 using an iTC-200 microcalorimeter (MicroCal, Inc.). **(B)** ITC binding curve for the titration of mutant $MLL1^{3762\text{-}3772}$_A3764R WIN motif to WDR5 using an iTC-200 microcalorimeter (MicroCal, Inc.). K_d: dissociation constants (µM). Related to Fig. 4.

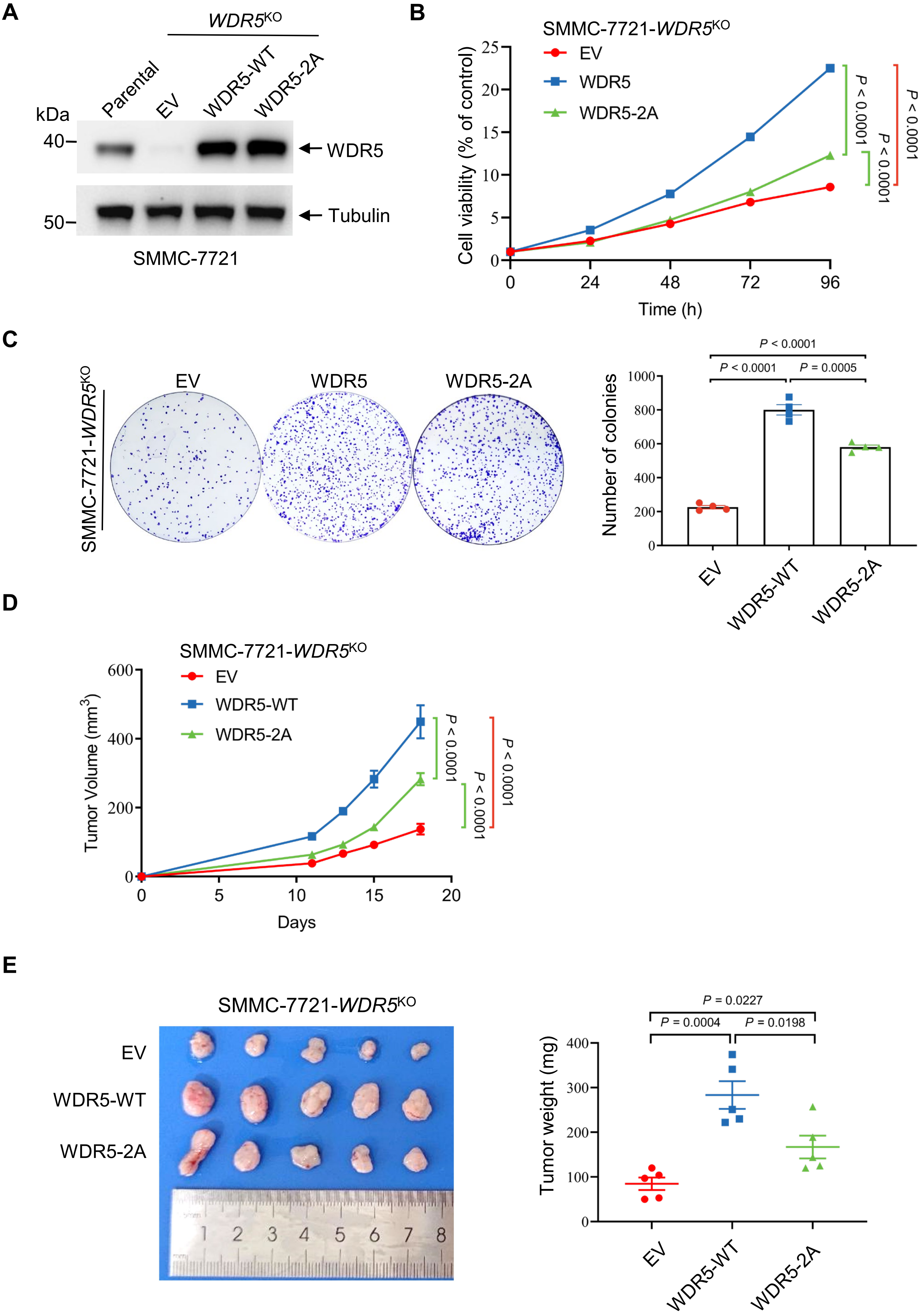

A
WDR5KO
Parental
EV
WDR5-WT
WDR5-2A
kDa
40
50
WDR5
Tubulin
SMMC-7721
B
SMMC-7721-WDR5KO
EV
WDR5
WDR5-2A
Cell viability (% of control)
Time (h)
P < 0.0001
P < 0.0001
P < 0.0001
C
SMMC-7721-WDR5KO
EV
WDR5
WDR5-2A
Number of colonies
P < 0.0001
P < 0.0001
P = 0.0005
EV
WDR5-WT
WDR5-2A
D
SMMC-7721-WDR5KO
EV
WDR5-WT
WDR5-2A
Tumor Volume (mm3)
Days
P < 0.0001
P < 0.0001
P < 0.0001
E
SMMC-7721-WDR5KO
EV
WDR5-WT
WDR5-2A
Tumor weight (mg)
P = 0.0227
P = 0.0004
P = 0.0198
EV
WDR5-WT
WDR5-2A

Fig. S8 The interaction between WDR5 and PTENα-NTE SSSRRSS motif is required in cancer progression. (A) The efficiency of rescue of *WDR5*^{KO} SMMC-7721 cells by ectopic expression of wild-type WDR5 or its mutant WDR5-2A (F133A/F263A). SMMC-7721 *WDR5*^{KO} cells were transfected with empty vector (EV), lentiviruses encoding wild-type WDR5, or its mutant WDR5-2A, followed by Western blotting of the indicated proteins. Pre-stained protein marker: ThermoFisher, 26616. **(B, C)** Disruption of the interaction between PTENα and WDR5 impaired tumor cell growth and tumorigenic capacity. CCK8 assays **(B)** and colony formation assays **(C)** were used to determine the proliferation and tumorigenic capacity of these cells. **(D, E)** Disruption of the interaction between PTENα and WDR5 destroyed the promotion of tumorigenesis by PTENα. These cells were subcutaneously injected into nude mice (1.5×10^6 cells per mouse; n = 5 mice per group). Tumor volumes were measured at different days **(D)**. On day 18, tumors were harvested, photographed **(left)**, and weighed **(right) (E)**. The experiments were repeated three times (twice for animal experiments) independently with similar results, and the results of one representative experiment were shown. For **B-E**, data represent means ± s.e.m. Statistical significance was determined by two-way ANOVA **(B, D)** or two-tailed unpaired *t*-test **(C, E)**. Full and uncropped Western blots are provided in Supplemental Material.

Table S1 Primer sequences

Protein fragments	Sequences
E. coli expression constructs[a]	
WDR5$^{1\text{-}334}$	5': ttgtatttccagggcATGGCGACGGAGGAGAAGAA<br>3': agtctgcagggtacctcaGCAGTCACTCTTCCACAGTT
WDR5$^{22\text{-}334}$	5': ttgtatttccagggcTCCGCCACTCAGAGCAAGCC<br>3': agtctgcagggtacctcaGCAGTCACTCTTCCACAGTT
PTENα-NTE$^{1\text{-}173}$	5': ttgtatttccagggcCTGGAACGCGGAGGGGAGGC<br>3': agtctgcagggtacctcaATCCGGCAGGCGATGGCTGA
PTENα$^{1\text{-}576}$	5': ttgtatttccagggcCTGGAGCGGGGGGGAGAAGC<br>3': agtctgcagggtacctcaGACTTTTGTAATTTGTG
Mutants[b]	
WDR5 (D107A)	5': CTTCTTGTTTCTGCCTCAGcTGACAAAACC<br>3': gCTGAGGCAGAAACAAGAAGGTTAGAATCTG
WDR5 (F133A)	5': GGACACAGTAATTATGTCgcTTGCTGCAAC<br>3': gcGACATAATTACTGTGTCCCTTCAGGGTTTTCAGAC
WDR5 (F263A)	5': GAATGAGAAATACTGCATAgcTGCCAATTTC<br>3': gcTATGCAGTATTTCTCATTCTTGTGGCCAG
PTENα-NTE$^{1\text{-}173}$ (R119A)	5': CAACCGAGCAGCTCCCGCgcATCGAGCCAC<br>3': gcGCGGGAGCTGCTCGGTTGCAGGCTCTGG
PTENα-NTE$^{1\text{-}173}$ (R118/119A)	5': CAACCGAGCAGCTCCgcCgcATCGAGCCAC<br>3': gcGgcGGAGCTGCTCGGTTGCAGGCTCTGG
PTENα-NTE$^{1\text{-}173}$ (115-119-5A)	5': CAGAGCCTGCAACCGgccgcCgCCgcCgcaTCGAGCCAC<br>3': gcGgcGGcGgcggcCGGTTGCAGGCTCTGGGCCTGTTTTTC
PTENα-NTE$^{1\text{-}173}$ (R135A)	5': CAGGCCGCCGCTGAGgctGGCGCGAGCG<br>3': agcCTCAGCGGCGGCCTGACTCTGAACC
PTENα-NTE$^{1\text{-}173}$ (R144A)	5': GCCACCGCCAAAAGCgctGCCATCAGCA<br>3': agcGCTTTTGGCGGTGGCGCTCGCGCC
PTENα-NTE$^{1\text{-}173}$ (R118A)	5': CAACCGAGCAGCTCCgctAGATCGAGCC<br>3': agcGGAGCTGCTCGGTTGCAGGCTCTGG
PTENα-NTE$^{1\text{-}173}$ (R118G)	5': CAACCGAGCAGCTCCggcAGATCGAGCC<br>3': gccGGAGCTGCTCGGTTGCAGGCTCTGG
PTENα-NTE$^{1\text{-}173}$ (R118K)	5': CAACCGAGCAGCTCCaaaAGATCGAGCC<br>3': tttGGAGCTGCTCGGTTGCAGGCTCTGG
PTENα-NTE$^{1\text{-}173}$ (R118H)	5': CAACCGAGCAGCTCCcacAGATCGAGCC<br>3': gtgGGAGCTGCTCGGTTGCAGGCTCTGG
PTENα-NTE$^{1\text{-}173}$ (R118M)	5': CAACCGAGCAGCTCCatgAGATCGAGCC<br>3': catGGAGCTGCTCGGTTGCAGGCTCTGG
PTENα-NTE$^{1\text{-}173}$ (R118L)	5': CAACCGAGCAGCTCCctgAGATCGAGCC<br>3': cagGGAGCTGCTCGGTTGCAGGCTCTGG

PTENα-NTE$^{1-173}$ (R118E)	5': GCAACCGAGCAGCTCCgagAGATCGAGCCAC<br>3': ctcGGAGCTGCTCGGTTGCAGGCTCTGGGC
PTENα-NTE$^{1-173}$ (R118F)	5': GCAACCGAGCAGCTCCttCAGATCGAGCCAC<br>3': aaGGAGCTGCTCGGTTGCAGGCTCTGGGC
Mammalian expression constructs[a]	
WDR5$^{1-334}$	5': gatgacgacaagggtaccATGGCGACGGAGGAGAAGAAG<br>3': ggtttaaacgggccctcaGCAGTCACTCTTCCACAG
PTENα$^{1-576}$	5': gtcccagactacgcaCTGGAGCGGGGGGGAGAAGC<br>3': tatagaatagggcccTCAGACTTTTGTAATTTGTG
Quantitative RT-PCR	
NOTCH3	5': GACGTCAGTGTGAACTCCTC<br>3': CAGGTTGGTGCAGATACCATGA
TCF19	5': CGGCTTCTTGGCTGTCAAAC<br>3': GGGAGCCTCCAATCCGC
SLC12A5	5': GCAGGAGCCATGTACATCCT<br>3': CCATGCAGGTGAGCACACA
GAPDH	5': TGCACCACCAACTGCTTAGC<br>3': ACAGTCTTCTGGGTGGCAGTG

Note: a, the lower cases of these primers are the sequences used by seamless assembly cloning; b, the lower cases of these primers are the mutation sites.

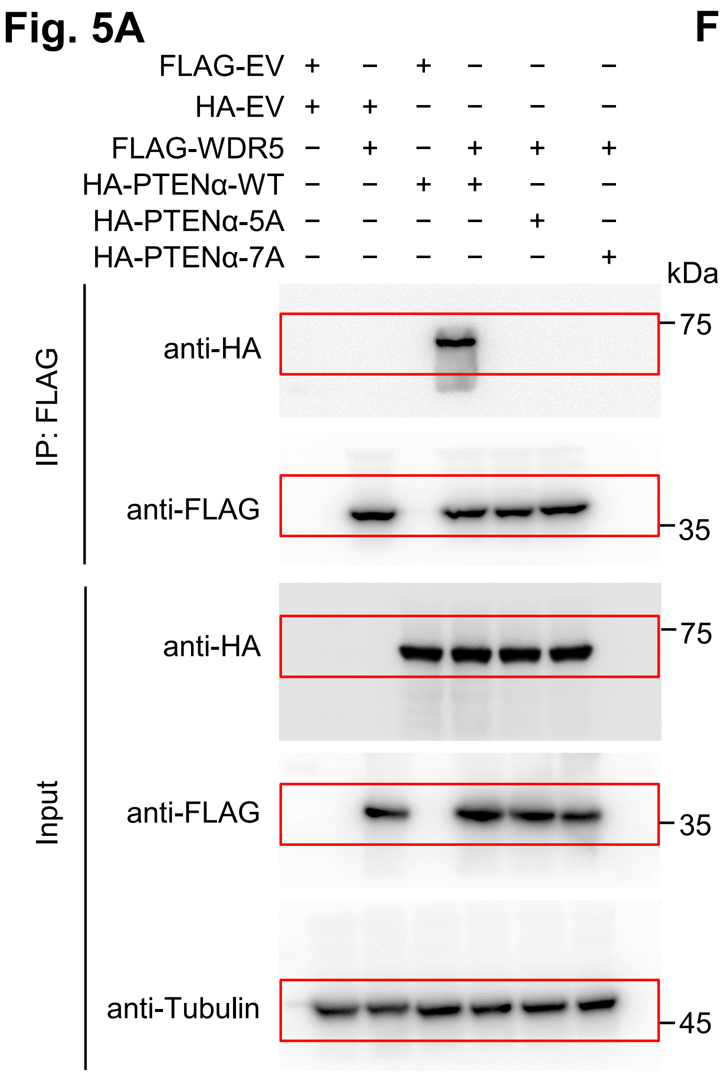

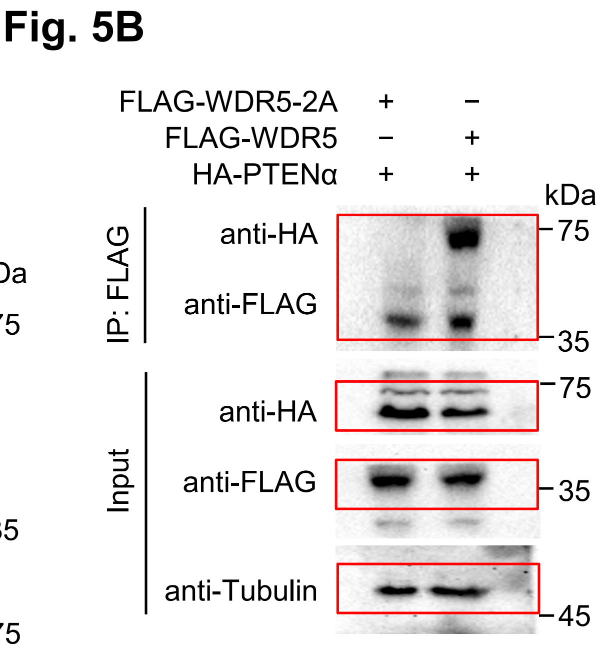

Full and uncropped Western blots for Fig. 5A and Fig. 5B.

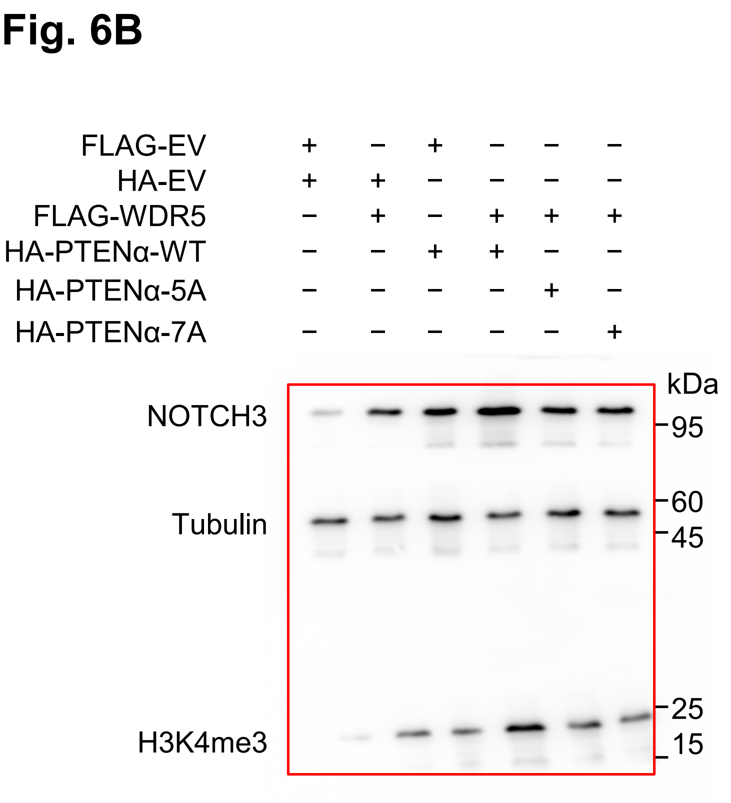

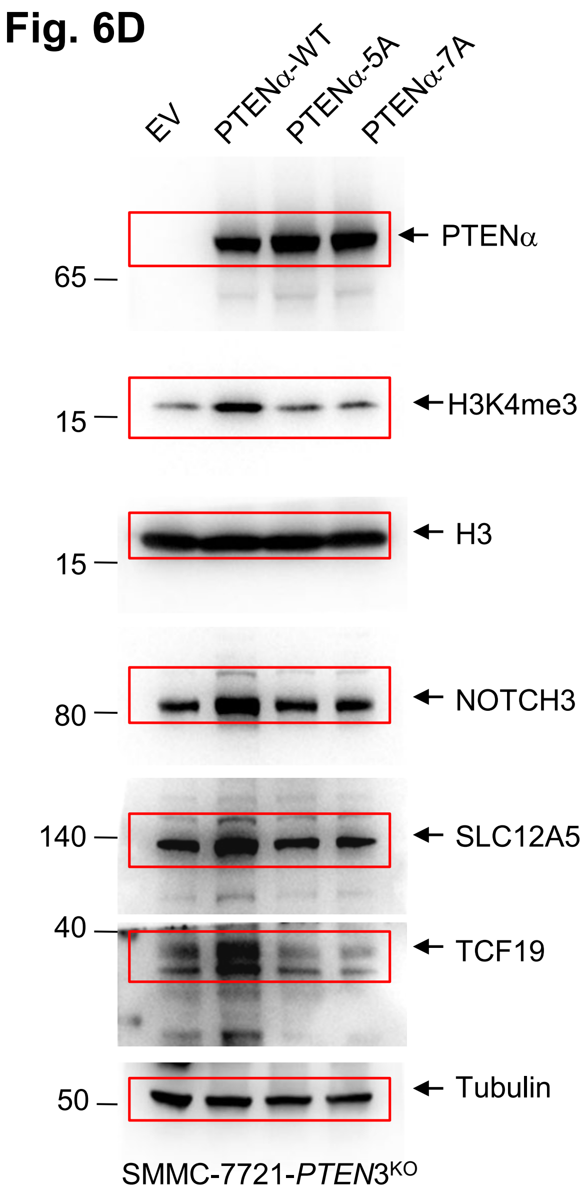

Full and uncropped Western blots for Fig. 6B and Fig. 6D.

Fig. 7A

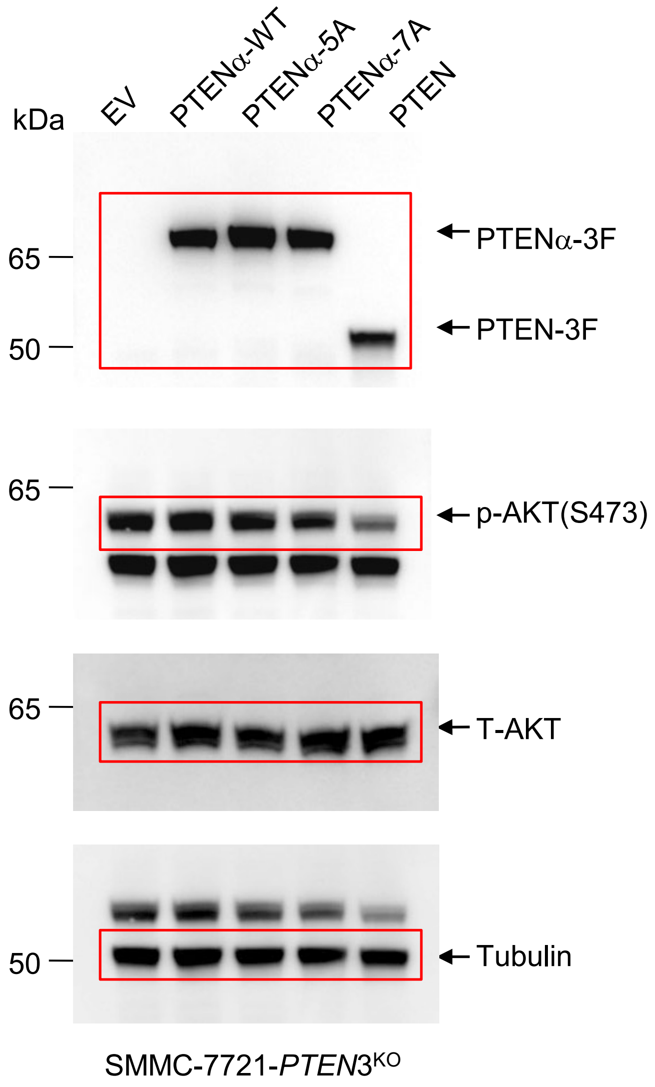

Fig. S8A

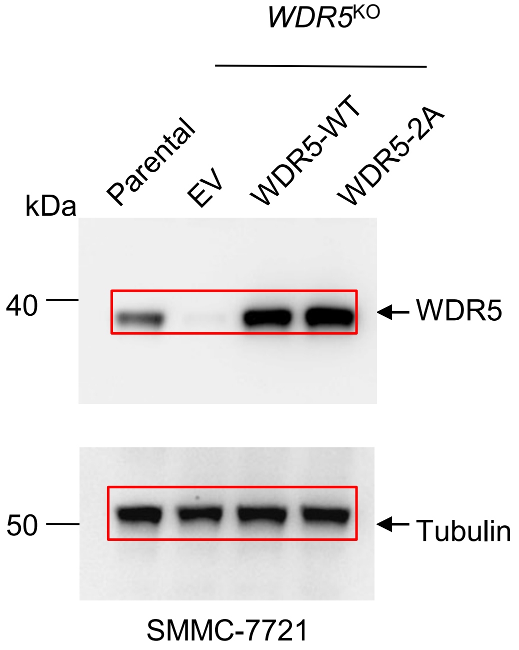

Full and uncropped Western blots for Fig. 7A and Fig. S8A.
